# Perfusion-guided sonopermeation of neuroblastoma: a novel strategy for monitoring and predicting liposomal doxorubicin uptake *in vivo*

**DOI:** 10.7150/thno.45903

**Published:** 2020-07-09

**Authors:** Aditi Bellary, Arelly Villarreal, Rojin Eslami, Quincy J. Undseth, Bianca Lec, Ann M. Defnet, Naina Bagrodia, Jessica J. Kandel, Mark A. Borden, Sumbul Shaikh, Rajiv Chopra, Theodore W. Laetsch, Lauren J. Delaney, Colette M. Shaw, John R. Eisenbrey, Sonia L. Hernandez, Shashank R. Sirsi

**Affiliations:** 1 Department of Biomedical Engineering, University of Texas at Dallas, Richardson, TX, USA.; 2 Department of Surgery, University of Chicago Medical School, Chicago, IL, USA.; 3 Biomedical Engineering, Mechanical Engineering, University of Colorado, Boulder, CO, USA.; 4 Department of Radiology, University of Texas Southwestern Medical Center, Dallas, TX, USA.; 5 Advanced Imaging Research Center, University of Texas Southwestern Medical Center, Dallas, TX, USA.; 6 Department of Pediatrics and Harold C. Simmons Comprehensive Cancer Center, University of Texas Southwestern Medical Center and Children's Health, Dallas, TX, USA.; 7 Department of Radiology, Thomas Jefferson University, Philadelphia, PA, USA.

**Keywords:** Sonoporation, sonopermeabilization, sonopermeation, neuroblastoma, quantitative contrast-enhanced ultrasound (qCEUS), image-guided drug delivery.

## Abstract

Neuroblastoma (NB) is the most common extracranial solid tumor in infants and children, and imposes significant morbidity and mortality in this population. The aggressive chemoradiotherapy required to treat high-risk NB results in survival of less than 50%, yet is associated with significant long-term adverse effects in survivors. Boosting efficacy and reducing morbidity are therefore key goals of treatment for affected children. We hypothesize that these may be achieved by developing strategies that both focus and limit toxic therapies to the region of the tumor. One such strategy is the use of targeted image-guided drug delivery (IGDD), which is growing in popularity in personalized therapy to simultaneously improve on-target drug deposition and assess drug pharmacodynamics in individual patients. IGDD strategies can utilize a variety of imaging modalities and methods of actively targeting pharmaceutical drugs, however *in vivo* imaging in combination with focused ultrasound is one of the most promising approaches already being deployed for clinical applications. Over the last two decades, IGDD using focused ultrasound with “microbubble” ultrasound contrast agents (UCAs) has been increasingly explored as a method of targeting a wide variety of diseases, including cancer. This technique, known as sonopermeation, mechanically augments vascular permeability, enabling increased penetration of drugs into target tissue. However, to date, methods of monitoring the vascular bioeffects of sonopermeation *in vivo* are lacking. UCAs are excellent vascular probes in contrast-enhanced ultrasound (CEUS) imaging, and are thus uniquely suited for monitoring the effects of sonopermeation in tumors.

**Methods**: To monitor the therapeutic efficacy of sonopermeation *in vivo,* we developed a novel system using 2D and 3D quantitative contrast-enhanced ultrasound imaging (qCEUS). 3D tumor volume and contrast enhancement was used to evaluate changes in blood volume during sonopermeation. 2D qCEUS-derived time-intensity curves (TICs) were used to assess reperfusion rates following sonopermeation therapy. Intratumoral doxorubicin (and liposome) uptake in NB was evalauted *ex vivo* along with associated vascular changes.

**Results**: In this study, we demonstrate that combining focused ultrasound therapy with UCAs can significantly enhance chemotherapeutic payload to NB in an orthotopic xenograft model, by improving delivery and tumoral uptake of long-circulating liposomal doxorubicin (L-DOX) nanoparticles. qCEUS imaging suggests that changes in flow rates are highly sensitive to sonopermeation and could be used to monitor the efficacy of treatment *in vivo*. Additionally, initial tumor perfusion may be a good predictor of drug uptake during sonopermeation. Following sonopermeation treatment, vascular biomarkers show increased permeability due to reduced pericyte coverage and rapid onset of doxorubicin-induced apoptosis of NB cells but without damage to blood vessels.

**Conclusion**: Our results suggest that significant L-DOX uptake can occur by increasing tumor vascular permeability with microbubble sonopermeation without otherwise damaging the vasculature, as confirmed by *in vivo* qCEUS imaging and *ex vivo* analysis. The use of qCEUS imaging to monitor sonopermeation efficiency and predict drug uptake could potentially provide real-time feedback to clinicians for determining treatment efficacy in tumors, leading to better and more efficient personalized therapies. Finally, we demonstrate how the IGDD strategy outlined in this study could be implemented in human patients using a single case study.

## Introduction

Neuroblastoma (NB) is the most prevalent extracranial solid tumor in infants and children. NB most often originates in the adrenal gland 1 and is diagnosed at a median age of 22 months. This malignancy is responsible for 15% of all childhood cancer deaths 2. High-risk NB is currently treated with maximally intense chemoradiotherapy, immunotherapy, and autologous stem cell transplant 3,4. Despite this, about half of the afflicted patient population will die of this disease 5. For survivors, this intense therapy is associated with long-term toxicities 6. For example, doxorubicin - which is a key component of multiagent chemotherapy for aggressive NB - is implicated in both acute and chronic adverse effects, including life-threatening cardiotoxicity 7. Therefore, methods of improving chemotherapeutic delivery are sorely needed to achieve better efficacy and reduce systemic toxicity.

One approach to this goal has been the development of nanomedicines to improve the circulation of drugs and payload into tumors. This strategy employs carrier vehicles with diameters between 10 and 200 nm to convey therapeutic drugs, which can either actively or passively target tumors *in vivo*. Currently, the most common method of targeting nanomedicines to cancers is by passive accumulation via the well-described Enhanced Permeability and Retention (EPR) effect 8. The EPR effect is commonly attributed to aberrant tumor vasculature, which is leaky (allowing ingress) and deficient in lymphatics (restricting drainage), causing long-circulating nanomaterials (between 50 and 750 nm) to gradually accumulate 9-11. This strategy was exploited in the first FDA-approved nanomedicine, liposomal doxorubicin (L-DOX) or Doxil™, which was developed to extend the circulating half-life of doxorubicin, enhancing both deposition of drug payloads within tumors and reducing drug cardiotoxicity 12. While this approach has been shown to function in specific cancer types, the use of nanomedicines such as L-DOX has not had the anticipated impact of significantly extending patient survival 13,14.

Furthermore, various studies have discovered the EPR effect to be highly dependent on the cancer type and stage 15. The extent of the EPR effect has been demonstrated to be highly exaggerated in xenograft tumor models, with less reliable efficacy in clinical cancers 16-18. Given this reality, novel strategies must be pursued to enhance the intratumoral accumulation of doxorubicin and other chemotherapeutics. More efficient delivery of circulating drugs could also cut down off-target adverse effects by reducing required systemic dosages for therapy 19. In these studies, we show that ultrasound-based IGDD holds promise as a means of achieving these therapeutic goals.

One promising method of overcoming reliance on the EPR effect utilizes the combination of ultrasound contrast agents (UCAs, also known as microbubbles) and focused ultrasound. Microbubbles are sound-sensitive gas-filled spheres (1-10 µm in diameter) that can mechanically disrupt cell bilayers or cellular gap junctions, allowing increased uptake of circulating drugs 20-23. Numerous studies have established that volumetric oscillation of the UCA in an ultrasound field can promote intracellular uptake of drugs by a membrane-permeabilizing process known as “sonopermeation” (also termed “sonoporation” or “sonopermeabilization”) 24-26. This technique has been utilized to achieve improved intracellular delivery of a wide variety of therapeutic compounds, including proteins 27, DNA 28-32, and nanoparticles such as liposomal doxorubicin 33, 34.

Despite these advances, nanoparticle delivery to cancer *in vivo* remains challenging. Physical co-treatments such as sonopermeation have progressively been adopted for applications such as blood-brain barrier (BBB) opening 35; Aryal *et al* doubled DOX concentrations in rat gliomas by co-injecting L-DOX with microbubbles and sonicating with focused US 36. Nevertheless, to date, only a few groups have employed sonopermeation *in vivo* to enhance L-DOX delivery to tumors beyond the brain. Theek *et al* showed that sonopermeation could improve intratumoral drug penetration, even in tumor models characterized by extensive stromal compartments and dense collagen networks 37. Tinkov *et al* illustrated that sonopermeation caused preferential uptake of doxorubicin in tumors, and noted a 12-fold increase in intratumoral drug concentration following sonopermeation 38. The majority of these studies used microscopy and tissue extraction procedures to quantify drug accumulation, quantifying tumor growth curves by physical caliper measurements 39. Thus, a major obstacle to implementing sonopermeation therapy in clinical practice is that *ex vivo* analysis currently functions as the only method to quantify drug uptake and monitor its bioeffects.

In the context of ultrasound-triggered microbubble destruction (UTMD), passive cavitation detection is being investigated as a technique to map acoustic emissions in order to calculate stable and inertial cavitation doses 40. This technique assumes that the risk of vascular damage correlates with increasing cavitation doses, but it doesn't accurately map the bioeffects associated with sonopermeation, since it focuses primarily on cavitation responses of the bubbles. Conversely, perfusion kinetics are dictated by the biology of the vasculature itself, and thus hold the potential to divulge the degree to which sonopermeation has altered vascular architecture and flow characteristics. We therefore set about to monitor sonopermeation efficacy *in vivo* using real-time perfusion imaging as feedback. Our study aimed to accomplish three objectives: (1) to demonstrate that sonopermeation can efficiently increase L-DOX uptake in tumor tissue (using a orthotopic neuroblastoma xenograft model), (2) to gauge whether perfusion kinetics can predict sonopermeation-induced efficiency of intratumoral drug uptake *in vivo* using quantitative contrast-enhanced ultrasound (qCEUS) imaging of the tumor vasculature, and (3) to explore molecular changes in NB in response to sonopermeation therapy, in order to better elucidate mechanisms of drug uptake.

In the course of this study, we show that contrast-enhanced focused ultrasound therapy can effectively increase nanoparticle uptake in experimental neuroblastoma by ~5-fold, and that qCEUS imaging can function as an effective predictor of sonopermeation efficiency *in vivo*. Specifically, our study results indicate that reperfusion rates (flow rates following a short fragmentation US pulse) are very sensitive to immediate changes in vascular permeability induced by bubbles. Changes in reperfusion rates immediately after sonopermeation increased by ~2-fold when using tumor-free Matrigel plug models, but decreased by ~2-fold when using neuroblastoma tumor models. Changes in total perfusion volume did not fluctuate significantly in either Matrigel plugs or neuroblastoma xenografts following sonopermeation. Although overall perfusion did not change significantly from pre- to post-treatment, we show that initial tumor perfusion can be a critical determinant of total chemotherapeutic uptake. Our results indicate that liposomal doxorubicin can effectively be delivered using focused ultrasound and microbubbles without damaging the integrity of blood vessels comprising the tumoral vascular network, which is essential for continued chemotherapeutic delivery to tumors.

We also demonstrate the biological effects of sonopermeation in neuroblastoma xenografts at 30 min and 24 h post-treatment. We show that ultrasound exposure with microbubbles causes discontinuous pericyte coverage and distended blood vessels, which likely leads to increased drug permeability and slower blood flow observed using qCEUS. Apoptotic effects of doxorubicin on tumor cells are apparent 24 h after sonopermeation using TUNEL staining, while no significant change in apoptosis is observed with sonopermeation alone. Our results suggest that anti-tumoral effects are instigated by drug treatment rather than the mechanical effects of microbubbles and ultrasound. Lastly, a case study in which image-guided qCEUS imaging was used to examine the effects of bubble destruction in a liver tumor, using a protocol analogous to the methodology used in neuroblastoma xenografts, has also been included to demonstrate the clinical feasibility of this strategy.

Together, this data serves as proof-of-principle that ultrasound image-guided therapy can provide a robust and rational approach to monitoring sonopermeation *in vivo*, with potential for predicting and controlling drug dosing in a clinical setting.

## Materials and Methods

### Preparation of Microbubbles

Microbubbles were formulated using a lipid film composed of 14.34 mg of 1,2-distearoyl-*sn*-glycero-3-phosphocholine (DSPC, 790.16 MW) and 5.66 mg of *N*-(methylpolyoxyethylene oxycarbonyl)-1,2-distearoyl-*sn*-glycero-3-phosphoethanolamine (DSPE-PEG2000, 2805.97 MW) (NOF Corporation, Tokyo, Japan), dissolved in chloroform (Sigma-Aldrich, St. Louis, MO). The lipid solution was evaporated for 48 h and then stored as a lipid film in a sealed scintillation vial at -20 °C. On the day of intended use, the 20 mg film was diluted to 2 mg/mL in a filtered mixture of 10% propane-1,2-diol (propylene glycol, 76.1 FW) (v/v), 10% propane-1,2,3-triol (glycerol, 92.09 FW) (v/v), and 10× phosphate buffer saline (PBS) diluted to 1× (Fisher Scientific, Waltham, MA). The lipid solution was heated to 65 °C on an Isotemp Heating Block and bath sonicated in a 1.9 L Ultrasonic Bath Sonicator (Fisher Scientific, Waltham, MA) until the lipid was completely suspended. Microbubbles were generated using probe micro-tip sonication (Branson 450 Ultrasonics Sonifier with microtip attachment, Emerson, St. Louis, MO) at 70% power under constant flushing with Decafluorobutane (PFB, 238 MW, FluoroMed LP, Round Rock, TX) for 10 s. The amalgamated lipid suspension was brought below the glass phase temperature and washed three times in a 10 mL Luer tip syringe (BD, Franklin Lakes, NJ) at 300 relative centrifugal force (RCF) for 3 min in a Bucket Centrifuge Model 5804R (Eppendorf, Hauppauge, NY) to concentrate and collect the bubbles, until the infranatant appeared clear. The microbubbles were characterized using a Multisizer 4e Coulter Counter (MS4, Beckman Coulter, Brea, CA) to determine size distribution and concentration.

### Liposome Formulation

A mock Doxil liposome was fabricated using a lipid film containing 7.56 mg of DSPC, 2.69 mg of cholesterol, and 2.44 mg of DSPE-PEG2000. The lipid solution was evaporated for 48 h and then stored as a lipid film in a sealed scintillation vial at -20 °C. On the day of intended use, the 10 mg film was diluted to 8 mg/mL with filtered pH 7.5 PBS and heated to 65 °C via bath sonication until the lipid became fully suspended. For the purpose of fluorescence microscopy, 1,1′-dioctadecyl-3,3,3′,3′-tetramethylindodicarbocyanine dye (DiD, 498.48 MW, 1 mM dilution, Sigma-Aldrich, St. Louis, MO) was added to the liposome solution at 2.1 µg per 1 mg of lipid and sonicated an additional 10 min.

### Matrigel Implantation

Optimization of image-guided drug delivery was initially performed in highly controllable Matrigel tumor models. Matrigel plugs are well-established angiogenesis models that have been extensively characterized 41,42. Matrigel plugs are formed by subcutaneous injection of a soluble basement membrane extract derived from the Engelbreth-Holm-Swarm tumor (Fisher Scientific, Atlanta, GA). Subsequent to injection, host endothelial cells rapidly migrate into the plug, and begin blood vessel formation. Blood vessel growth occurs relatively uniformly between plugs and without the complexities and type-specific heterogeneity that typically occur with different xenograft tumor models. Therefore, we reasoned these could function as excellent models for studying the effects of sonopermeation on perfusion kinetics in neovasculature. Prior to injection, frozen Matrigel was thawed overnight on ice. 12 μg human recombinant basic fibroblast growth factor (bFGF, Stemgent, Beltsville, MD) and 350 μg heparin were added to 10 mL Matrigel to stimulate blood vessel ingrowth. 1 mL of Matrigel mix was loaded into chilled 1 mL BD syringes using chilled 16-gauge needles. After loading, the needle was replaced with a 27-gauge needle and kept on ice until animals were prepared. No exogenous cells (tumor or endothelial) were used in Matrigel studies.

All animal experiments were approved by the UT Dallas or University of Chicago IACUC. Female CD-1 mice (Charles River, Wilmington, MA) were anesthetized with 2-3% isoflurane (Vedco, St. Joseph, MO) and restrained in the prone position. A shaved and disinfected region above the left kidney received 1 mL of chilled Matrigel solution subcutaneously, creating a spherical plug (1 mm^3^). At 14 days post-injection, mice were imaged with MB contrast agents to evaluate blood perfusion and tissues harvested for processing.

### Orthotopic NGP Tumor Model and Implantation

NGP cells are MYC-N amplified 43, and thus are a commonly used model for poor prognosis NB. They share many features of clinical NB such as histology, frequency, and location of metastatic lesions when renally implanted 44, as was done in nude athymic mice (Charles River, Wilmington, MA) to generate tumor models for this study. Mice were first anesthetized with inhalable isoflurane. After being positioned in a sterile environment, the entire right side of each mouse was cleaned with ethanol and painted with Betadine. A 3-5 mm diagonal incision was made with a scalpel blade in the direction of the ribcage atop the kidney. The underlying fascia was divided with scissors to expose the mouse's internal organs. The kidney was identified and delivered from the abdominal cavity, using forceps and a cotton-tipped applicator. A 27-gauge needle (of length 1.3 cm, BD Biosciences) fitted to a syringe containing 100 μL of cell suspension (1x10^6^ NGP cells in Phosphate Buffered Saline, Leibniz Institute DSMZ-GmbH, Braunschweig, Germany) was inserted into the kidney and its contents injected slowly. The kidney was then returned to the abdominal cavity. The incision was closed in layers with absorbable suture, followed by staples to close the skin. Mice were monitored daily to ensure complete recovery, and tumors were allowed to grow for 5-6 weeks (1-2 g weight) before ultrasound experiments were commenced.

### 3D Volume and Perfusion Imaging

3D imaging was performed by mounting a linear 15L8 transducer, equipped with the Acuson Sequoia 512 ultrasonography system (Siemens Medical Solutions, Erlangen, Germany), on a stepper motor, and sweeping it across the length of the tumor in 0.2 mm increments. Data was collected and subsequently analyzed using custom LabVIEW software. The resulting series of 2D section scans was combined in ImageJ to capture volumetric measurements (B-mode) and to map the vasculature in and around the tumor (CPS mode). Non-linear contrast images were acquired following a bolus injection of 5x10^7^ MBs in a total volume of 100 µL, administered via tail vein catheterization.

### Perfusion-Guided Monitoring of Drug Uptake in Matrigel Plugs

**Figure 1:**
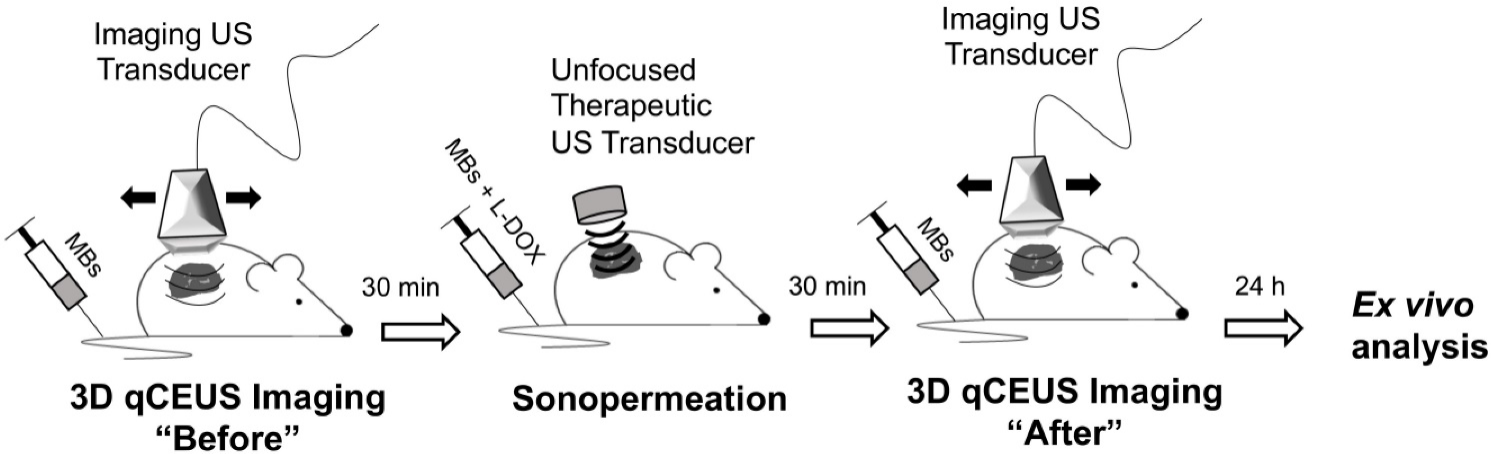
Cartoon depiction of workflow for unfocused ultrasound experiments. Mice bearing tumor-free Matrigel plugs were imaged (15 MHz) in 2D and 3D prior to sonopermeation to establish a baseline of tumor perfusion using a bolus of 5x10^7^ MBs. Next, microbubbles and drug were systemically introduced via tail vein injection and subcutaneous tumors were sonopermeated with a handheld therapeutic ultrasound transducer (1 MHz) using a high concentration of MBs (1x10^9^ MBs in 500 µL). 30 min post-sonopermeation, tumor-free Matrigel plugs were imaged again to assess changes in the vasculature. Finally, tissue was excised 24 h after sonopermeation and *ex vivo* analysis was performed to quantify FITC-Dextran uptake.

Mice bearing Matrigel plugs were 3D imaged prior to sonopermeation to assess the initial level of perfusion; Matrigel plugs were utilized to mimic tumors as they are a good model for angiogenesis. A high dose of microbubbles (1x10^9^ total MBs) was combined either with 25 mg/kg L-DOX (Doxoves^©^, FormuMax, Sunnyvale, CA) or FITC-Dextran (2 kDa, 5 mg/mL in PBS) and systemically introduced via tail vein injection. Subcutaneous tumor-free Matrigel plugs were sonopermeated with a handheld therapeutic ultrasound machine (SoundCare Plus, Austin, TX) at 0-3 W/cm^2^ (1 MHz, 10% duty cycle, alternating 5 seconds on and 5 seconds off) for 10 min. 30 min post-sonopermeation, the tumors were again 3D imaged to evaluate changes in the vasculature. Finally, Matrigel, contralateral kidney, liver, and heart tissue were excised 24 h after sonopermeation and *ex vivo* analysis was performed (Figure 1).

### Sonopermeation *In vivo* Using Focused Ultrasound Application

**Figure 2:**
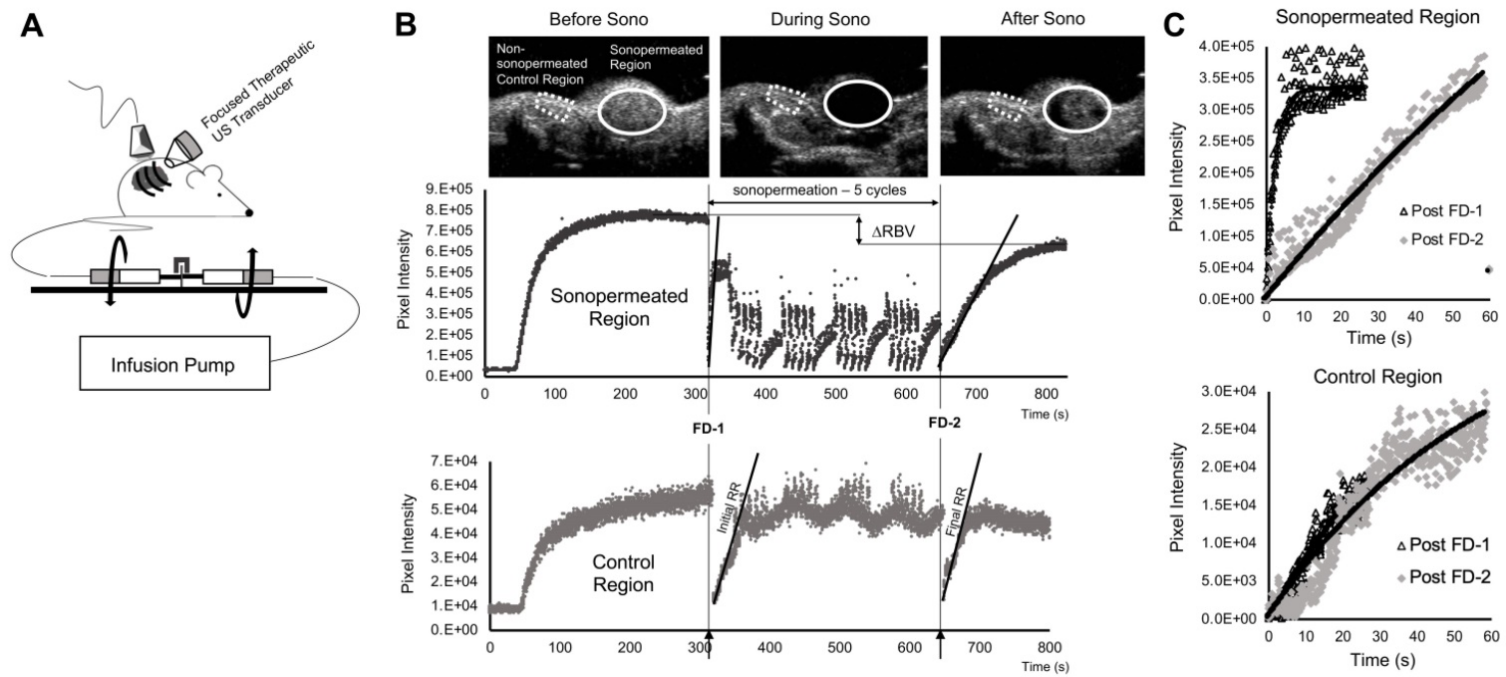
Perfusion-guided sonopermeation of neuroblastoma tumor models using qCEUS imaging techniques to monitor vascular changes. (A) A rotating syringe pump was used to administer constant infusions over long periods of time during sonopermeation experiments. (B) Screen captures from the clinical ultrasound scanner illustrate non-linear 2D imaging of a neuroblastoma tumor sonopermeated using 1x10^9^ MBs. The selection of appropriate ROIs encompassing the sonopermeated focal zone (solid white line) and a designated control region (dashed white line) at the periphery of the tumor has been indicated. The series of ultrasound images show contrast enhancement before sonopermeation, followed by focused ultrasound triggered microbubble destruction *in vivo* during four sonopermeation treatment cycles, culminating in MB recovery within the tumor space post-sonopermeation; the accompanying time-intensity curves (TICs) - corresponding to the “treated” and “untreated” ROIs - have been split into three matching stages. (C) MB reperfusion after the initial (FD-1 = 300 s) and final (FD-2 = 630 s) flash-destruction pulses were fitted to an exponential model and the resultant curves were compared to assess sonopermeation-mediated changes in perfusion kinetics.

A custom lens and cone system was developed to elevate pressure in the focal zone (~ 2 MPa peak negative pressure, Supplementary Figure 1) and was affixed to the therapeutic ultrasound probe. A commercial infusion pump (Kent Scientific, Torrington, CT) was coupled to a custom 3D printed rotating syringe platform (similar to Kaya *et al* 45), designed to evenly disperse the microbubbles in solution, ensuring that sustained infusions occurred at a fixed concentration throughout the duration of microbubble administration (Figure 2A). Pre- and post-sonopermeation perfusion kinetics were obtained in a single 10 min infusion: 1x10^9^ microbubbles were combined with 100 µL of DiD liposomes and brought up to a total volume of 500 µL with sterile saline. Microbubbles flowed into the tumor space at a constant rate of 50 µL/min and were allowed to reach steady-state over a period of 5 min, after which a flash-destruction pulse (MI = 1.9) was applied to clear all bubbles from the imaging plane. Microbubbles were then given 30 s to re-circulate, following which tumors were sonopermeated (3 W/cm^2^) on/off five times for 5 s each. This procedure was repeated for four cycles, interspersed by 30 s gaps to permit replenishment. At the 10.5 min mark, the tumor was hit with a final flash-destruction pulse (Figure 2B).

As described by Wei *et al* 46, microbubble reperfusion following flash-destruction is governed by the exponential equation:

y = A (1 - e^-βt^)

Perfusion recovery curves generated from CPS data were fit to this form in the LabVIEW software and plotted on the same set of axes (Figure 2C). Measures of the relative blood volume (RBV = A) and the rate of reperfusion (RR = β) were extracted and compared pre-treatment and post-treatment in the sonopermeated region and an area outside the tumor (designated as the internal “control region”) for each mouse.

### Tumor and Matrigel Excision

Mice were sacrificed 24 h post-sonopermeation by exsanguination to remove drug remaining in circulation. Prior to exsanguination, mice were anesthetized using 5% isoflurane. After confirming depth of anesthesia by toe pinch, animals were exsanguinated by intracardiac perfusion of 1% paraformaldehyde (for IHC of mice sonopermeated without drug) or cold saline (for drug quantification). This procedure was performed by inserting a syringe with 20 mL of solution into the left ventricle and cutting the right atrium prior to flushing out the mouse's circulatory system. All Matrigel plugs/tumors were surgically excised for *ex vivo* processing immediately after perfusion.

### FITC Dextran Quantification in Matrigel Plugs

Drug uptake in Matrigel plugs was evaluated using a procedure similar to a previously reported study by Prewett *et al* 47. Matrigel plugs were removed and transferred to tubes containing 0.5 mL of Dispase reagent (Becton Dickinson). Plugs were incubated for 1 h at 37 °C and then stored overnight at 4 °C. The plugs were then tip sonicated using a microtip sonicator (Branson) at 30% power until a homogenous slurry was obtained. Sterile saline was added to the tubes, which were then vortexed and centrifuged at 2000 rpm for 5 min. The supernatant was separated and the accumulated FITC-Dextran was quantified by measuring the fluorescent properties of the 2 kDa FITC (488/515 nm ex/em) on a fluorescent microplate reader (Synergy H1 Biotek, Boston, MA). A standard curve of known FITC-Dextran concentrations was used to determine total uptake in the Matrigel plugs after normalizing to plug weight.

### Doxorubicin Quantification in Matrigel Plugs and NGP Tumors

Doxorubicin quantification used a similar approach adopting a combination of two methodologies by Bing *et al* 48 and Head *et al* 49. Matrigel plugs or tumors were excised, weighed, and snap frozen. Sections of tissue were weighed (typically 50-100 mg) in 1.5 mL centrifuge tubes with a cell lysis buffer (consisting of 0.25 M sucrose, 5 mM Tris-HCl, 1 mM MgSO_4_, 1 mM CaCl_2_ pH 7.6) and 100 µL ceramic beads (MO BIO Laboratories, Carlsbad, CA). The tubes were placed in a vial mixer (Bristol-Meyers Squibb, New York, NY) for 45 s to homogenize the tissue. In order to establish standards of known doxorubicin measurements in homogenates of Matrigel plugs and tumors, untreated tissue was mixed with 2 µL of 10 mg/mL doxorubicin (Sigma Aldrich) stock dissolved in DMSO and homogenized as described above. Spiked homogenates were then serially diluted with extraction buffer. The readings of untreated plugs or tumor samples without doxorubicin were considered zero. The untreated samples with known concentrations of doxorubicin were used in every plate, in order to accurately and consistently quantify doxorubicin accumulation in treated tissue. Untreated and treated homogenized samples (200 µL) were placed in 1.5 mL microcentrifuge tubes, with acidified isopropanol solution: 100 µL of 10% (v/v) Triton X-100 (Sigma Aldrich), 200 µL of water, and 1 mL of acidified isopropanol (0.75 N HCl, Sigma Aldrich). Samples were stored overnight at -20 ºC to extract the doxorubicin. The next day, the microcentrifuge tubes were warmed to room temperature, vial mixed for 45 s, centrifuged at 2,000 g for 15 min in a mini vortexer, and stored at -80 ºC until analysis. The standard curve generated by spiking tissue with known quantities of doxorubicin was run side by side with experimental samples to quantify uptake per gram of tumor or Matrigel. Doxorubicin was also quantified in heart, liver, and contralateral kidney tissue using the same method.

### Fluorescent Drug Uptake in NGP Tissue Sections

For NGP tumors, whole tissue sections were used to evaluate intratumoral accumulation of doxorubicin and DiD tracer liposomes to assess spatial distribution within the tumor. Tumors were embedded in Tissue-Tek® optimum cutting temperature (O.C.T.) compound (Electron Microscopy Services) over dry ice until frozen, then stored at -80 ºC until cryosectioned. 5 μm thick cryosections were visualized without mounting media on a confocal microscope (Marianas or SP2, Zeiss).

### Histology and Immunohistochemistry

Some tumors and organs were fixed in 4% paraformaldehyde overnight, dehydrated in 70% ethanol and paraffin embedded. 5 μm sections were used to stain for the apoptosis marker terminal deoxynucleotidyl transferase dUTP nick end labeling (TUNEL, ApopTag, Millipore, following the manufacturer's instructions), the selective pericyte marker alpha-smooth muscle actin (αSMA) 47 (LabVision Actin, ThermoFisher Scientific), the endothelial markers Endomucin (Santa Cruz Biotechnology), IsolectinB4-AlexaFluor488 (ThermoFisher Scientific), and hematoxylin and eosin. Whole H&Es, αSMA, Endomucin, and TUNEL tissues were scanned in a CRi Pannoramic Midi 20x Whole Slide Scanner (3DHistech). Data and images were analyzed using Pannoramic Viewer software (3DHistech). DOX and DiD were quantified on fresh frozen tumors stored in OCT and then cut into 5 μm sections, using a Marianas Confocal Microscope (Zeiss) at 561 nm laser (L-DOX) and 640 nm laser (DiD) wavelengths. At least eight images at a 40x magnification of three tumors per group were taken at random by focusing on the DAPI channel. Quantification was performed in ImageJ, using the mean grey area of 16-bit images, normalized by total DAPI area to account for variations in intratumoral structures, such as a large blood vessel without tumor cells. Figures were then processed in Adobe Illustrator cc 2018 (Adobe). In order to measure vascular caliber (blood vessel diameter), we used tumor sections stained against a marker of the endothelium, the innermost layer of cells in blood vessels, known as Endomucin. NGP tumors immunostained against Endomucin were scanned, and images analyzed using the Pannoramic Viewer software (3DHISTECH). Taking this endothelial marker as an indicator of the vascular wall, we defined the vascular caliber as the longest axis spanning the inside of Endomucin stained endothelial cells, using the “Create Distance Measurement Annotation” on Pannoramic Viewer software. One hundred measurements of vessel caliber were created from each tumor section in randomized locations within each tumor, three tumors were analyzed per group, and statistical analysis performed in Prism (GraphPad) using a Wilcoxon Signed Rank Test (Supplementary Figure 5). Lectin- and αSMA-positive cells were quantified from fluorescent images taken at 10x magnification on an Axioscop2 (Zeiss), and using the threshold tool in ImageJ. Statistical analysis was performed with Student's T-test in Excel (Microsoft). Four pictures per tumor section for at least three sections were taken from fresh frozen samples, excluding kidney areas and non-viable tumor areas.

### Sonopermeation in Human Patients

Human imaging data was collected as part of an ongoing clinical trial using sonopermeation for sensitizing hepatocellular carcinoma to radioembolization therapy (NCT #03199274). This study is approved by the Food and Drug Administration (IND #126,768) and the Institutional Review Board of Thomas Jefferson University. All patients provided informed consent prior to participation. Imaging was performed using an S3000 scanner with a 6C1 probe and flash-replenishment and non-linear imaging packages (Siemens Healthineers, Mountain View, CA) by a sonographer possessing over ten years of experience.

For contrast agent infusions, 5 mL of activated Optison (GE Healthcare) was suspended in 50 mL of saline and infused through an 18- to 20-gauge angiocatheter placed in a peripheral arm vein at a rate of 120 mL/h. The tumor midline was first imaged using Cadence Pulse Sequencing (MI = 0.06) for at least 45 s until complete enhancement of the tumor and adjacent liver was observed. Following complete enhancement, the patient was asked to temporarily halt respiration. Sonopermeation was performed using a 4 s destructive pulse from the imaging transducer (MI = 1.13 at 1.5 MHz, transmitting 2.3 µs pulses at a pulse repetition frequency of approximately 100 Hz), which is applied to all microbubbles in the field of view. Following sonopermeation, Optison reperfusion was imaged at the tumor midline for at least 10 s. This flash-replenishment sequence was repeated for at least 5 cycles at the tumor midline after which full intratumoral microbubble destruction was obtained by manually sweeping through the tumor during the 4 s destructive sequences for the remainder of the infusion. Following sonopermeation, patients were monitored for 30 min and data extracted in DICOM format for off-line processing.

## Results

### Sonopermeation Using Unfocused Ultrasound Does Not Increase Doxorubicin Uptake in Matrigel Plugs

**Figure 3:**
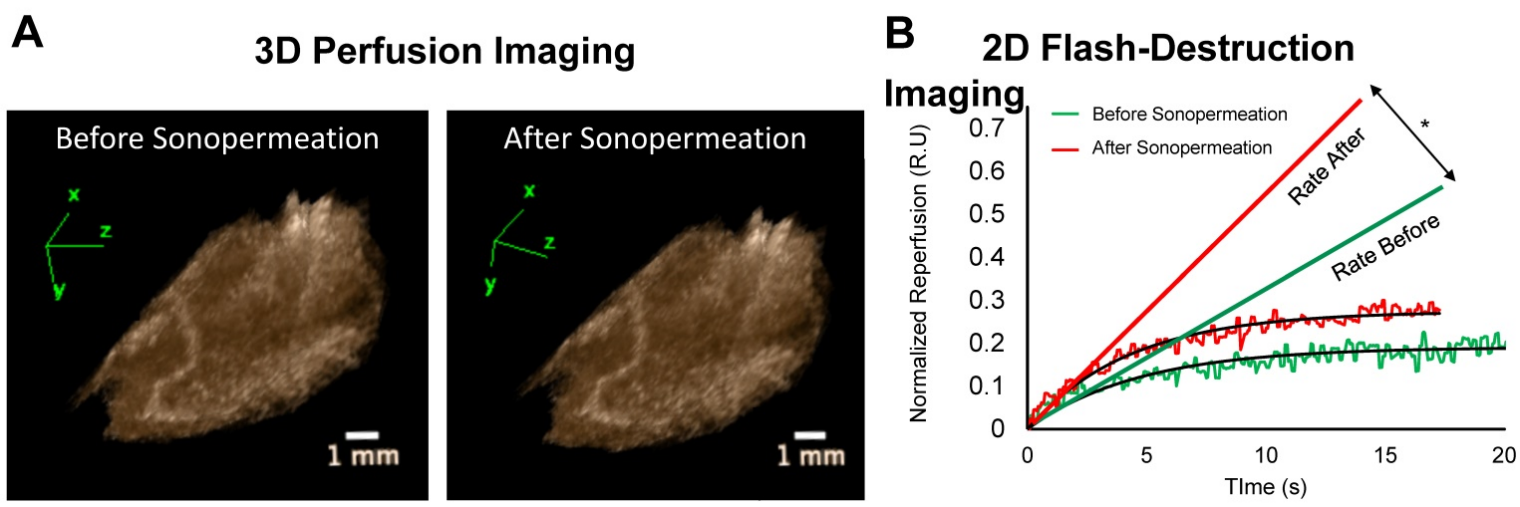
(A) Examples of 3D reconstructions of Matrigel plugs generated from interpolating a series 2D non-linear contrast images taken pre- and post-unfocused sonopermeation reveal no overall changes in relative blood volume or level of perfusion. (B) Examples of 2D flash-destruction imaging performed before (green) and after (red) unfocused sonopermeation show an increase (p<0.05) in the rate of microbubble reperfusion in Matrigel plug models. * Indicates statistical significance in the difference between pre- and post-sonopermeation rates as compared to untreated Matrigel plugs.

Image-guided studies were performed by delivering 2 kDa FITC-Dextran molecules to tumor-free Matrigel plugs to test whether an (unfocused) commercial therapeutic ultrasound system can improve small molecule uptake. We applied 0 to 3 W/cm^2^ ultrasound intensities (which corresponded to 0-0.6 MPa PnP, Supplementary Figure 1). In these studies, 2D and 3D contrast enhanced ultrasound (CEUS) imaging was performed immediately before and 30 min after sonopermeation therapy to assess changes in overall perfusion and perfusion kinetics (Figure 3A-B). We did not detect significant changes in perfusion volume in the Matrigel plugs using the unfocused system (Figure 3A). Conversely, there was a statistically significant increase in the rate of reperfusion after unfocused sonopermeation at 2 and 3 W/cm^2^ (Figure 3B, red vs. green line).

**Figure 4:**
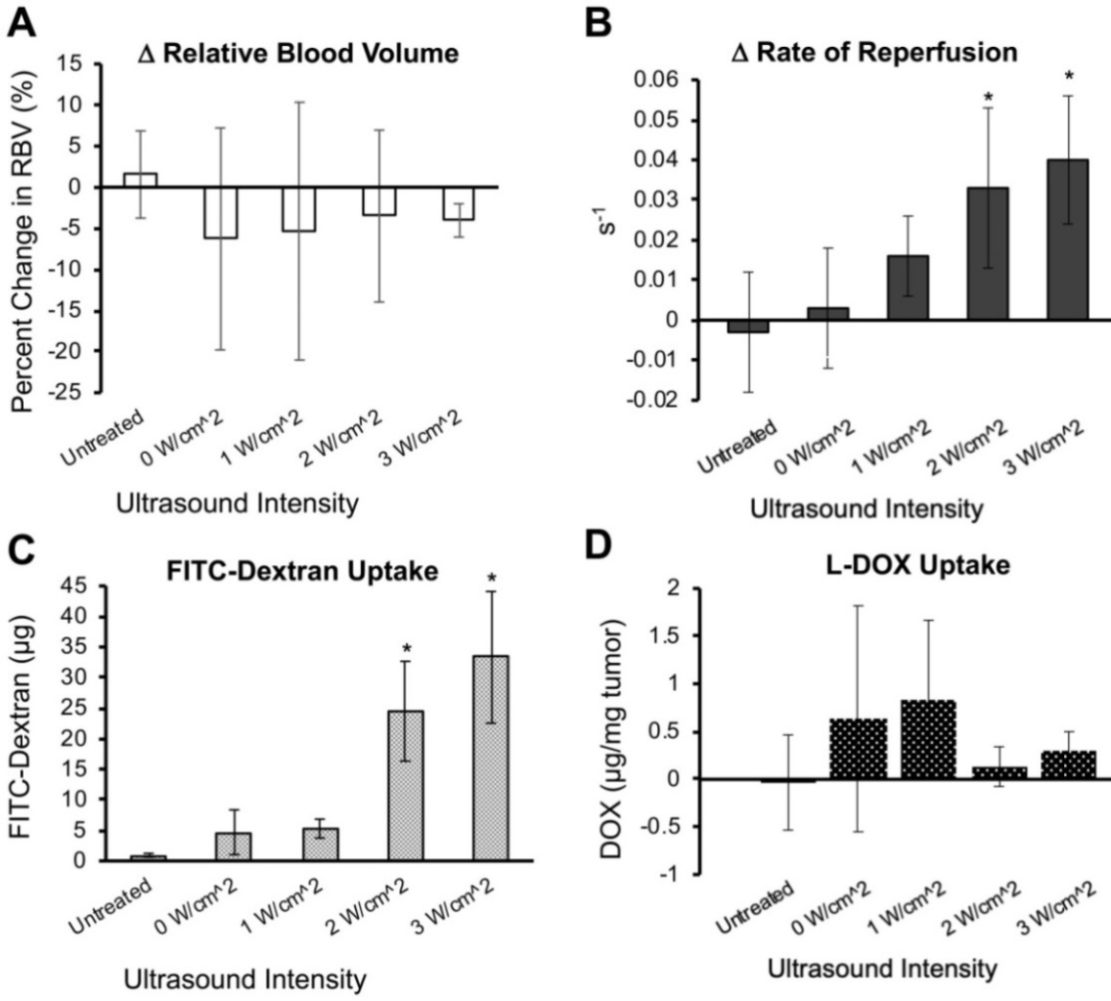
Effect of unfocused sonopermeation on tumor perfusion and drug uptake. Mice assigned to the untreated group received neither microbubbles nor FITC-Dextran/DOX nor ultrasound, whereas the 0 W/cm^2^ group received microbubbles co-injected with FITC-Dextran/DOX without ultrasound. (A) Percent change in the relative blood volume before and 30 min post-sonopermeation from 3D qCEUS. (B) Change in reperfusion rates before and after sonopermeation. (C) Small molecule (2 kDa) FITC-Dextran uptake in Matrigel plugs 24 h post-sonopermeation. (D) Doxorubicin uptake delivered using liposomal L-DOX 24 h post-sonopermeation. Ultrasound intensities from 0-3 W/cm^2^ correspond to 0-0.6 MPa PnP. N = 4-5 mice per group. * Indicates p<0.05 compared to the untreated group.

Next, we further characterized the effects of unfocused ultrasound on perfusion and drug uptake using Matrigel plugs. 3D contrast enhancement in the Matrigel plugs did not show significant changes in the relative blood volume (RBV) following sonopermeation therapy at any intensity level (Figure 4A). However, reperfusion rates measured using destruction-replenishment imaging showed a significant increase in reperfusion velocity with increasing ultrasound power (Figure 4B). While there is a linear increase in the rate of reperfusion with increasing ultrasound pressure, only ultrasound intensities of 2 W/cm^2^ and 3 W/cm^2^ had statistical increases in reperfusion rates above baseline (Figure 4B, 0.03 and 0.04 s^-1^ respectively, p<0.05). Interestingly, the increase in reperfusion rate correlated with small-molecule FITC-Dextran uptake, which showed recovery of 24.5 and 33.3 µg FITC-Dextran per gram of Matrigel tissue at 2 W/cm^2^ and 3 W/cm^2^ respectively (Figure 4C, p<0.05).

After demonstrating increased FITC-Dextran delivery to Matrigel plugs, the methodology was repeated using liposomal doxorubicin (L-DOX). Despite increased FITC-Dextran uptake, there were no significant changes in levels of doxorubicin in excised Matrigel plugs following the same unfocused sonopermeation experiments (Figure 4D). We rationalized that this is likely due to the size of the L-DOX molecules (~80 nm), which would not extravasate as effectively as free drug in circulation. In the next section, we detail our approach for improving sonopermeation efficiency of liposomes using focused ultrasound. In all subsequent experiments, only uptake of large molecule L-DOX or liposomes was investigated.

### Sonopermeation Using Focused Ultrasound Increases Liposome and Doxorubicin Uptake in Matrigel Plugs

**Figure 5:**
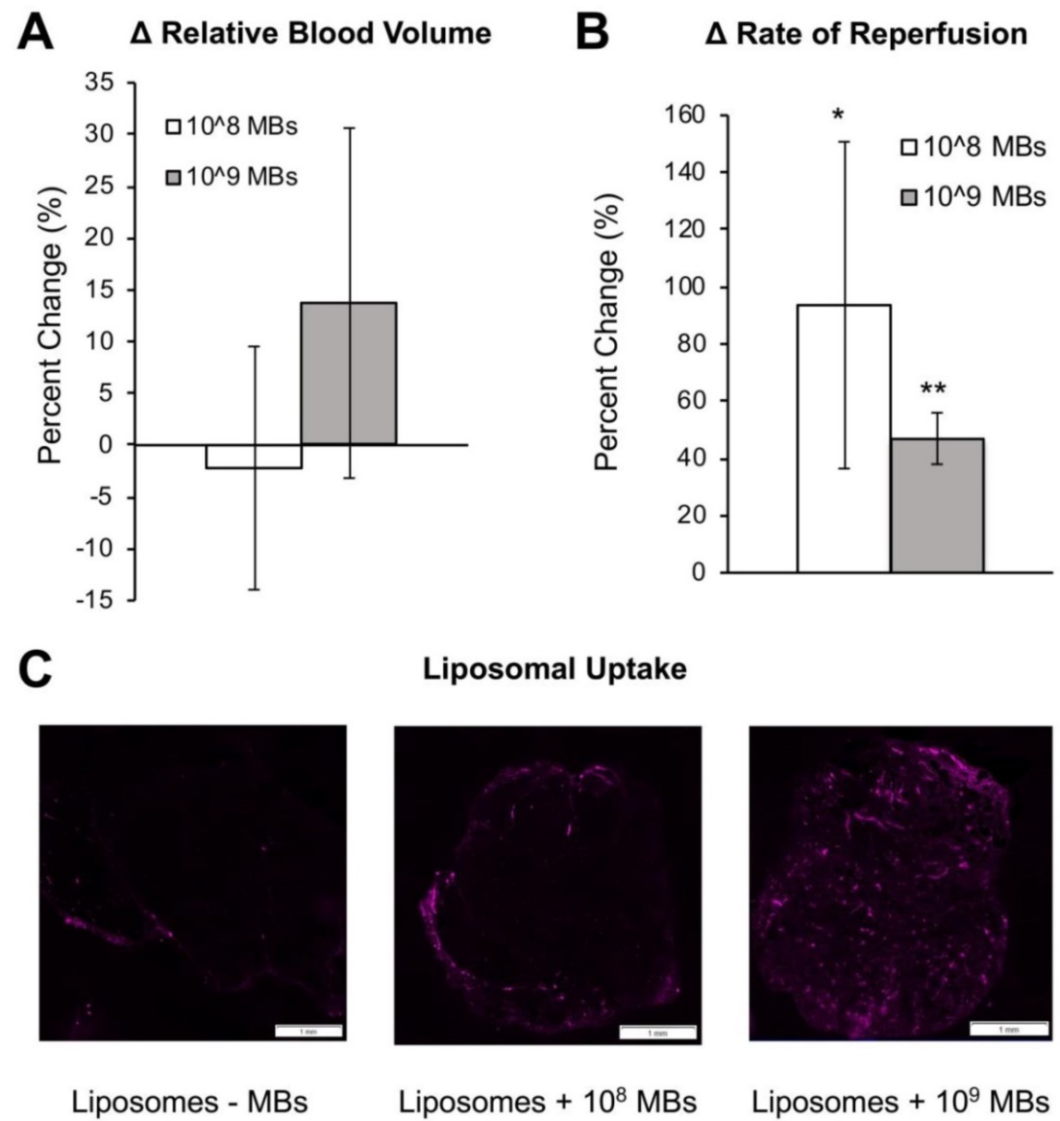
Evaluation of focused ultrasound treatment on qCEUS parameters and DiD Liposome uptake in Matrigel plugs. (A) Changes in RBV from baseline (before sonopermeation) using two different concentrations of MBs co-injected with DiD labeled liposomes. (B) Change in reperfusion rate after sonopermeation using the same tumors. Values were calculated by selecting an ROI in the sonopermeated focal zone and normalizing against non-sonopermeated control ROIs outside of the tumor area, as described in Figure 2. A paired Student's T-test was performed with N = 3 mice per group. * Indicates p<0.05 and ** indicates p<0.01 and reflect that these groups are significantly different than the baseline value. (C) Effect of MB concentration on nanoparticle accumulation. Sections of sonopermeated and non-sonopermeated Matrigel plugs were visualized on a whole slide scanner (Olympus VS120 Virtual Slide Microscope).

In order to achieve higher levels of ultrasound application that would facilitate larger molecule uptake in tumors, we utilized a custom focusing lens attached to the therapeutic ultrasound system to generate pressures up to 2.35 MPa (Supplementary Figure 1) within the tumor space. We then tested whether focused ultrasound could increase larger particle (80 nm liposomes) uptake in Matrigel plugs. In order to facilitate *ex vivo* detection, the liposomes were labeled by incorporating DiD in the bilayer. Focused ultrasound was applied at a maximal pressure of 2 MPa using either 10^8^ or 10^9^ total MBs together with liposomes diluted in PBS. Similar to unfocused ultrasound (Figure 4A), total perfusion in the Matrigel plugs remained unchanged before and after sonopermeation (Figure 5A), but reperfusion rates increased significantly in the focal region where ultrasound was applied (Figure 5B). Liposomal uptake was detected in Matrigel plug cross sections by DiD fluorescence (Figure 5C, purple) and greater accumulation was qualitatively observed in the sonopermeated tumors as compared to the untreated negative control tumors (Figure 5C, middle and right panel vs. left panel).

Furthermore, *ex vivo* analysis of Matrigel plugs showed increased levels of RBC extravasation at elevated pressures of up to 2 MPa using focused ultrasound, potentially indicative of egress of 4-7 μm diameter particles (murine erythrocytes are 4-7 μm; Supplementary Figure 3, arrows).

**Figure 6:**
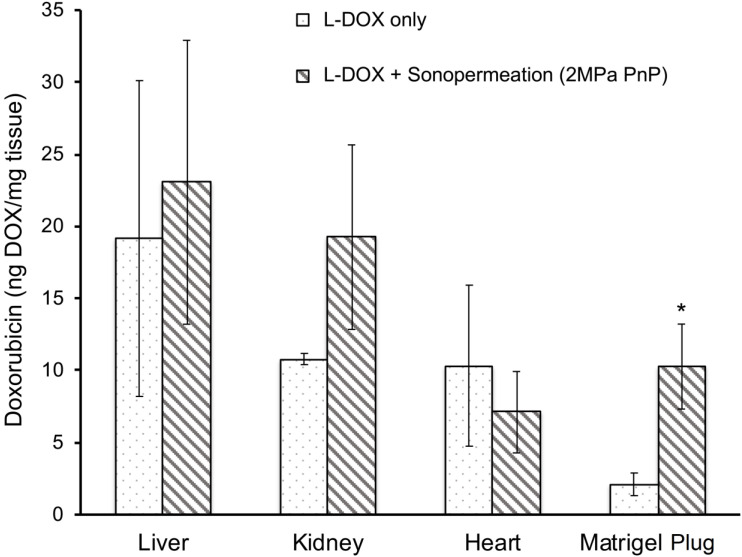
Focused ultrasound increases drug uptake in Matrigel plugs. A 5-fold increase in L-DOX accumulation was achieved in Matrigel plugs sonopermeated with a high dosage of microbubbles (10^9^ MBs in 500 µL) as compared to their non-sonopermeated counterparts, accompanied by no statistically significant difference in other organs, suggesting that sonopermeation drove more L-DOX selectively into the tumor without simultaneously increasing uptake in the heart, all without increasing the dosage of administration. N = 5 mice per group. * Indicates p<0.05.

Next, we asked whether sonopermeation with focused ultrasound would increase L-DOX uptake in Matrigel plugs. When sonopermeated and non-sonopermeated mice were compared, a 5-fold increase in L-DOX accumulation was observed in Matrigel plugs accompanied by no statistically significant change in any of the other organs analyzed (Figure 6, p<0.05). Histological analysis confirmed this tissue extraction data, wherein greater liposomal uptake was noted in Matrigel tumors alone (Figure 5C, data for heart, liver and kidney not shown). While only one L-DOX concentration was tested in this study, future studies that examine a lower range of concentrations are warranted to demonstrate that lower dosages can achieve an equivalent therapeutic response to standard L-DOX treatments.

### Initial Degree of Vascularity Influences the Extent to Which Sonopermeation Enhances Doxorubicin Uptake

**Figure 7:**
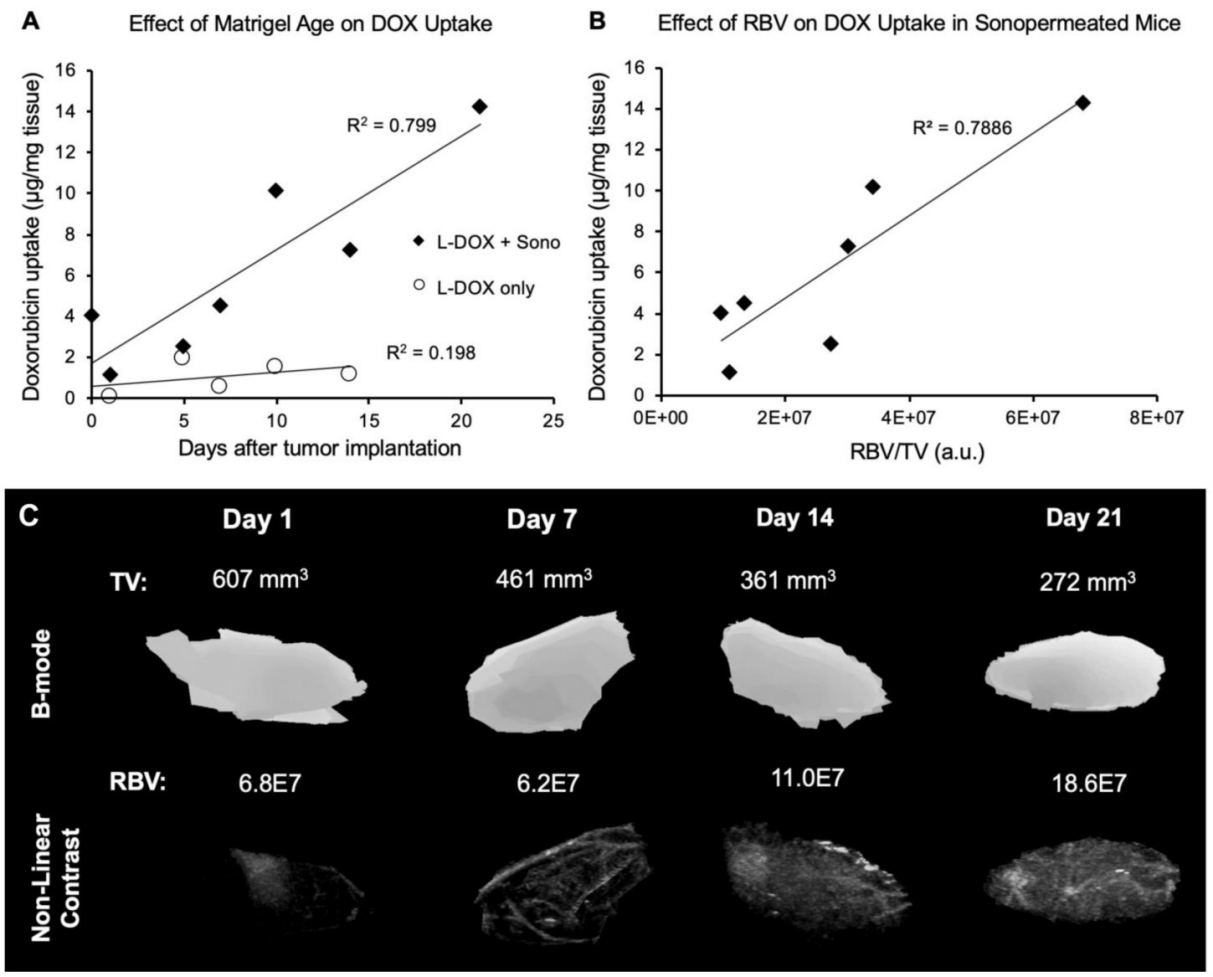
Effect of Matrigel age and perfusion on doxorubicin uptake. (A) Sonopermeation efficiency increases with Matrigel plug maturity (black diamonds). No effect of Matrigel age on doxorubicin uptake is observed without sonopermeation (white circles). (B) Normalized relative blood volume (RBV) measurements from qCEUS imaging of Matrigel plugs show a similar increasing trend in doxorubicin uptake with increased perfusion. (C) Examples of 3D qCEUS measurements of tumor volume (TV) and perfusion (RBV) from four sonopermeated Matrigel plugs. Matrigel plugs are reabsorbed over time causing a decrease in TV while RBV increases, thus enhancing the effectiveness of sonopermeation.

Since no change in relative blood volume was distinguishable pre- and post-sonopermeation (Figures 3A & 5A), we addressed the question of whether perfusion volume does, in fact, affect sonopermeation efficiency. Given that the number and size of blood vessels will increase over time in a Matrigel plug, we evaluated whether the number of days post-implantation, and thus vascularization levels, affect DOX uptake. We found that vascularity alone does not facilitate DOX uptake (Figure 7A, white circles). However, when sonopermeation is combined with greater perfusion volume, there is a linear increase in L-DOX uptake (Figure 7A, black diamonds), implying that the level of tumor perfusion may serve as a good predictor of drug accumulation. Increases in vascularity were confirmed in sonopermeated mice with qCEUS imaging (Figure 7B) by using the relative blood volume (RBV, measured in arbitrary pixel units) and normalizing to tumor volume (TV, mm^3^). Example images of perfusion within tumor-free Matrigel plugs at varying time points post-implantation are shown in Figure 7C. Matrigel plug sizes tend to shrink over time as they are reabsorbed in the body while blood vessel growth continues to increase with time, likely leading to more efficient doxorubicin uptake per unit volume.

### Sonopermeation Increases Vascular Permeability in Neuroblastoma

**Figure 8:**
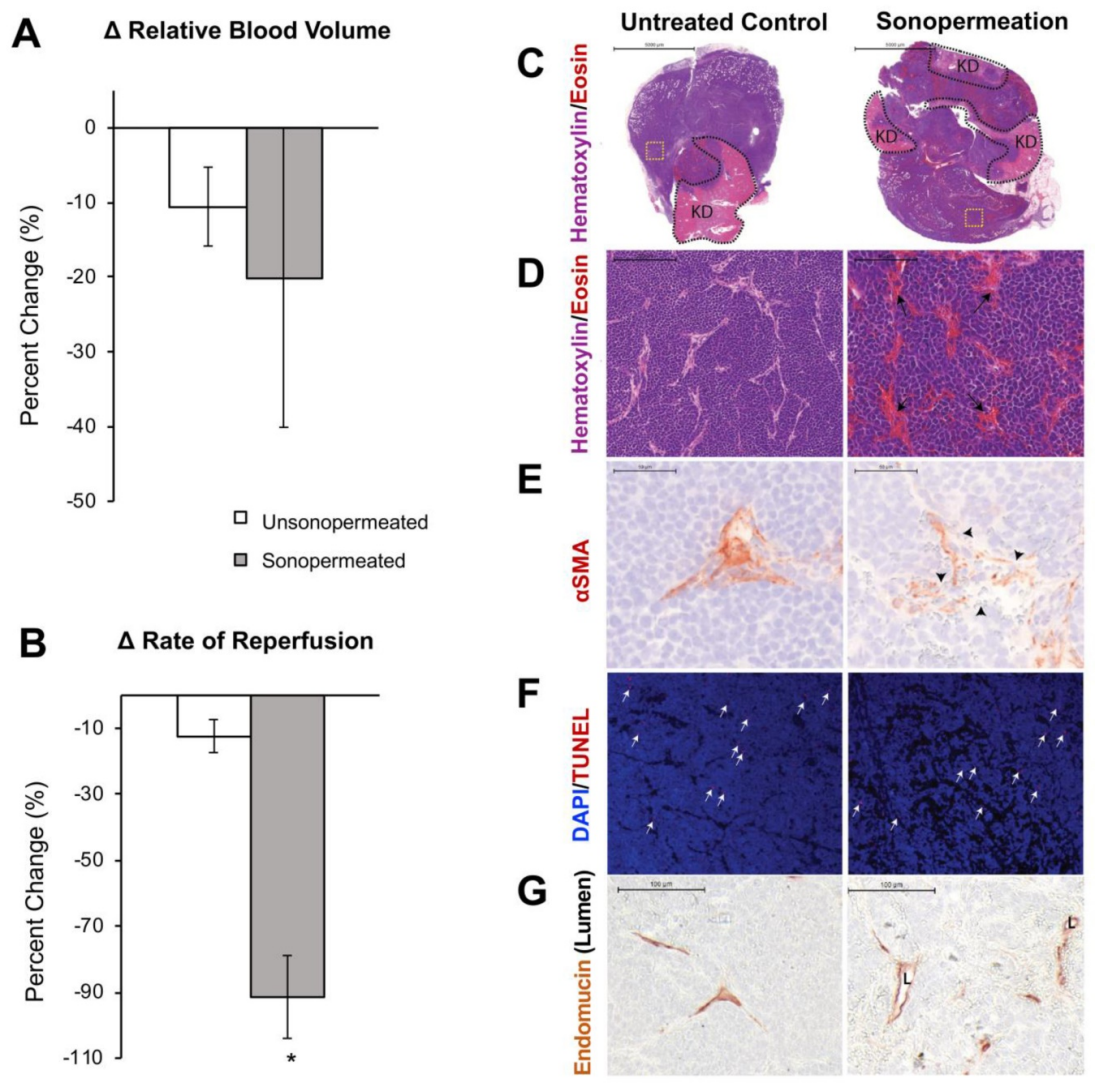
Effect of sonopermeation alone (no L-DOX) on qCEUS parameters and NGP tumoral vasculature 30 min post-treatment. (A) Change in RBV after sonopermeation within the sonopermeated tumor compared to unsonopermeated control region outside the tumor (see Figure 2 for ROI selection). (B) Change in RR after sonopermeation using the same ROIs. (C-G) *Ex vivo* analysis of NGP tumors harvested 30 min after sonopermeation alone (right column) or following no treatment (Untreated Control, left column). (C) Hematoxylin (blue) and eosin (pink-red) (top and middle rows) with remaining kidney (KD, light pink) highlighted by the dotted black line (top row) revealed no change in cell death as a result of sonopermeation (homogeneous blue staining). (D) 20x magnification (middle row) of an area away from normal kidney (yellow dotted square in top row) shows increased blood vessel diameter (black arrows) in the treated tumors compared to untreated controls. (E) Sonopermeation led to discontinuous pericyte coverage (black arrowheads) illustrated by interrupted alpha-smooth muscle actin immunostain (αSMA, third row). (F) TUNEL staining (red, white arrows) was used to detect cell death, which was minimally observed in untreated or sonopermeation alone mice. (G) Sonopermeation alone increases blood vessel dilation. Lumens (“L”) were measured using Pannoramic Viewer; the longest distance between Endomucin immunostained cells was measured, and over 100 measurements of randomized locations per tissue were analyzed (see Supplementary Figure 5). N = 3 mice per group.

Next, we turned our focus from Matrigel model tumors to orthotopic human NB xenografts. Similar to the Matrigel model, quantification of changes in relative blood volume divulged no statistically significant change in the level of perfusion between the non-sonopermeated and sonopermeated regions in xenografts (Figure 8A). However, a significant decrease (79±23%, p<0.01) in reperfusion rate was observed in the sonopermeated region compared to the untreated control region (Figure 8B). In order to further characterize the effects of sonopermeation alone, we analyzed tumor histology 30 min after treatment. Hematoxylin and eosin (H&E) revealed sonopermeated tumor overall histology and architecture were comparable to that of untreated controls (Figure 8C). The lighter, pink areas demarcate remnant kidney tissue (Figure 8C, first row “KD”), unaltered by sonopermeation, and blue staining overall indicates no loss of tumor viability or architectural changes (Figure 8C). Upon higher magnification, we found that sonopermeation did induce vascular dilation versus untreated controls (Figure 8D, black arrows). In order to confirm whether sonopermeation affected the vascular lumen, we immunostained tumors against the endothelial marker Endomucin. We then measured the longest axis between Endomucin-stained endothelial cells on opposing vessel walls, and validated that sonopermeation significantly increased vascular dilation (Figure 8G; median diameter of 15.9 µM for controls compared to 21.5 µM for sonopermeation, RankSum 653 in controls vs. 3003 in sonopermeation, p<0.0001). Pericytes contribute to capillary diameter and vascular permeability through their direct contact with endothelial cells; we interrogated the effect of sonopermeation on pericytes using the marker αSMA (reviewed by Bergers and Song 50), used to identify mature pericytes in experimental neuroblastoma 51. Immunostaining of sonopermeated tumors revealed discontinuous pericyte coverage of endothelial cells, consistent with increased vascular dilation and permeability (Figure 8E, black arrowheads). In order to determine whether changes in pericyte morphology were due to pericyte death, we analyzed the apoptosis marker TUNEL. TUNEL staining revealed no change in pericyte apoptosis in the sonopermeated tumors (Figure 8F, white arrows), or in the number of TUNEL positive αSMA cells (data not shown). Finally, we observed an increase in extravasated red blood cells adjacent to discontinuous pericytes as a result of sonopermeation (Figure 8E, 8G). Together, this data suggests that sonopermeation itself (at the prescribed settings in this study) can increase vascular permeability in NGP tumors without damaging the vasculature.

### Sonopermeation Increases Doxorubicin Uptake in Orthotopic NB Xenograft Tumors and Increases Tumor Apoptosis

**Figure 9:**
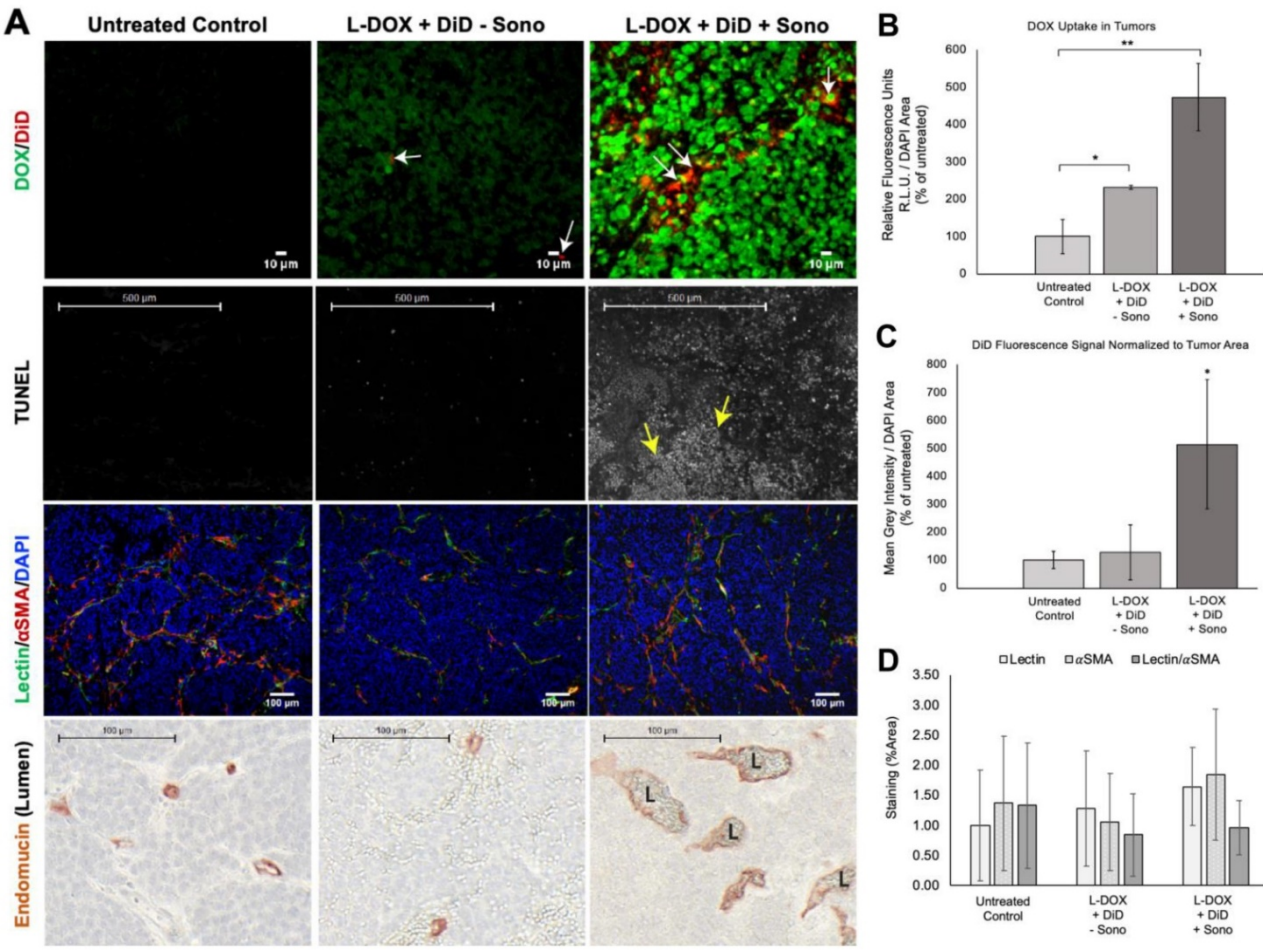
Evaluating drug uptake and vasculature changes in NGP tumors 24 h after sonopermeation. (A) *Ex vivo* evaluation of NGP tumors using histology and immunostaining to evaluate levels of doxorubicin and liposomal DiD uptake, cell death (TUNEL), lectin, αSMA, and Endomucin (lumen). Untreated tumors (left column) were compared with tumors given L-DOX and DiD liposomes only (no sonopermeation, middle column), and sonopermeated tumors (right column). Low magnification of the apoptosis marker TUNEL reveals that sonopermeation with L-DOX led to large clusters of apoptotic tumor cells at 24 h (second row, white dots, yellow arrows) although no observable changes in blood vessel (lectin) or pericyte coverage (αSMA) were observed (third row). Sonopermeation caused enlargement of vascular lumens (marked L) in the presence of L-DOX: lumens were measured using Pannoramic Viewer, the longest distance separating Endomucin immunostained cells was measured, and over 100 measurements of randomized locations per tissue were analyzed (see Supplementary Figure 5). (B) Quantification of doxorubicin in NGP tumors calculated by average fluorescent intensity within the tumors. Image intensities are ~2 and 5-fold higher for L-DOX/DiD only and sonopermeated tumors with L-DOX/DiD respectively. Untreated controls show a small level of background signal due to tissue autofluorescence. (C) Comparison of liposome uptake in NGP tumors using average DiD fluorescent intensity. Infusion of L-DOX without sonopermeation did not alter DiD uptake in tumors compared to the untreated controls. Sonopermeation increased DiD presence in the tumors approximately 5-fold. (D) Quantification from low magnification (10x) images reveals no significant changes in endothelial marker lectin or pericyte marker αSMA. N = 3 mice per group. * Indicates p<0.05 and ** indicates p<0.01.

NGP tumors receiving L-DOX and DiD liposomes without sonopermeation had 231±33% increase in L-DOX fluorescent intensity compared to untreated ones (Figure 9B, p<0.05). DiD signal, however, was barely detectable in this group, only 127±96% of the untreated control (Figure 9C, p=ns). In contrast, sonopermeated tumors had 476±89% L-DOX compared to untreated controls and 204±38% more L-DOX than non-sonopermeated mice (Figure 9B, p<0.01); we found L-DOX to accumulate in the nuclei of tumor cells that had undergone sonopermeation (Figure 9A, top right panel, green). In parallel with this finding, sonopermeated tumor DiD fluorescence was 514±232% of untreated controls (Figure 9C, p=0.04) and 404±183% of non-sonopermeated mice (Figure 9C, p=0.056); mice treated with L-DOX and DiD liposomes without sonopermeation had no difference in DiD compared to untreated controls (Figure 9C, p=ns). This data suggests increased vascular permeability as a result of sonopermeation. Next, we quantified the vascular lumen 24 h post-sonopermeation by measuring the intervening length between cells lining the vasculature on either side of the vessel, using the Endomucin marker (Figure 9A, bottom row, brown color). Indeed, the vascular caliber was significantly larger in sonopermeated tumors than in untreated controls (median diameter of 15.9 µM for controls versus 29.8 µM for sonopermeation; Rank Sum 653 vs. 4072 respectively, p<0.0001), demonstrating that the vascular dilation observed 30 min post-sonopermeation (Figure 8G) not only persisted but increased 24 h later (p<0.01). Meanwhile, lumens of tumors receiving L-DOX without sonopermeation were not different from untreated controls (Figure 9A, bottom row, middle panel).

Next, we analyzed the effects of enhanced, sonopermeation-induced L-DOX delivery to tumors at the cellular level. Histological analysis revealed large clusters of TUNEL positive tumor cells in sonopermeated tumors (Figure 9A, middle row, white dots, yellow arrows). Although L-DOX alone increased the number of apoptotic tumor cells compared to untreated controls, the pattern of apoptotic cells remains scattered throughout the tissue, suggesting that many tumor cells escaped L-DOX treatment in the absence of sonopermeation. Sonopermeation, on the other hand, revealed large areas of tumor cell death owing to the effects of DOX after a single treatment, as is evident in this example (Figure 9A, middle right panel, yellow arrows). In sum, our data suggests that sonopermeation increases tumoral DOX uptake by increasing vascular permeability.

### Sonopermeation in Clinical Testing

**Figure 10:**
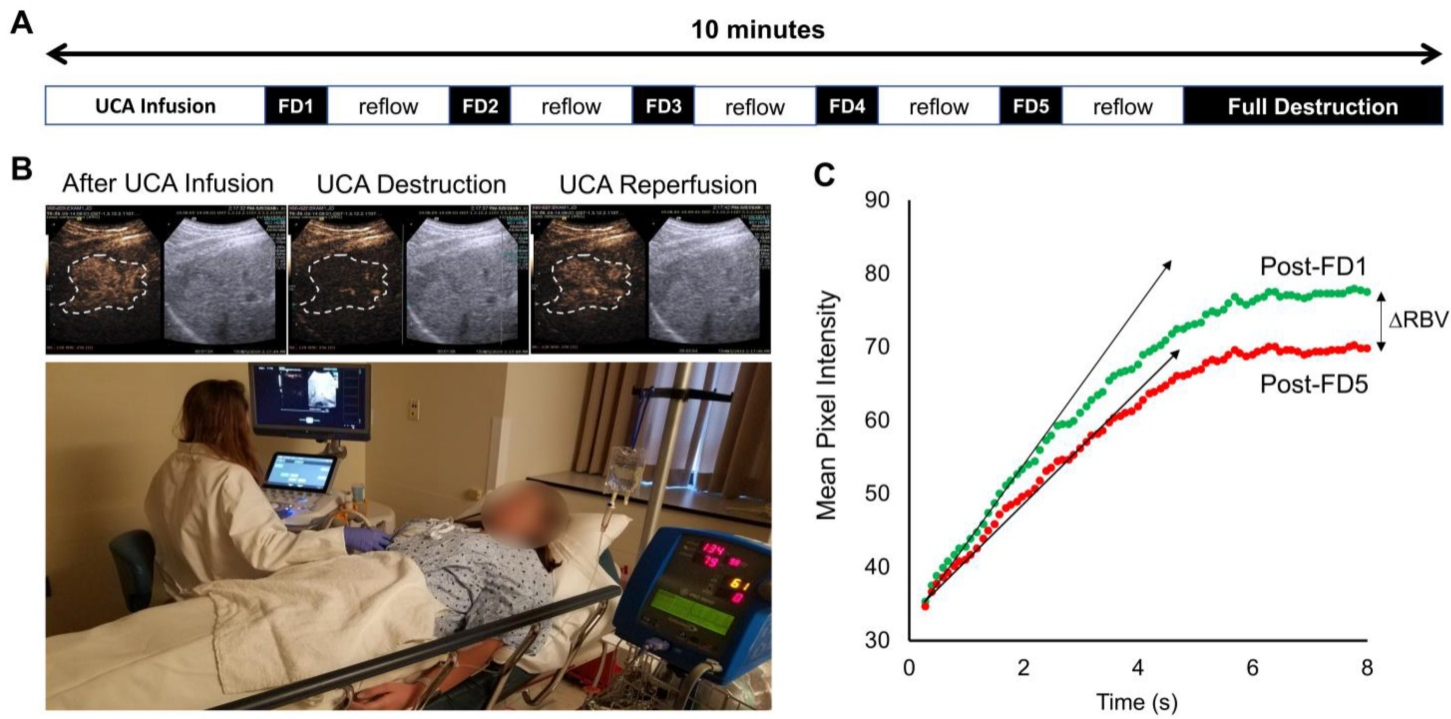
Sonopermeation in a clinical setting. The above images constitute an example of how image-guided sonopermeation can be accomplished in patients using a single continuous infusion of ultrasound contrast agents. (A) Summary of methodology for performing imaging and flash-destruction cycles followed by full destruction of contrast agents. (B) Representative images of contrast agent infusion, contrast agent destruction, and reflow in hepatocellular carcinoma (outlined in white). (C) Mean pixel intensities immediately following the first (green) and last (red) flash destruction pulse.

Here, we present the results from a single patient sonopermeation experiment (Figure 10). Similar to NGP xenograft studies which show slower reperfusion following 4 cycles of sonopermeation, qCEUS also shows a slight decrease in relative blood volume (RBV) following sonopermeation (Figure 10C); however the UCA replenishment times in this study were much shorter than what was used for NGP xenograft studies, thus full reperfusion may not be observed. Although the procedures and results between human and animal testing were similar, there are several key differences between the experiments noted in the discussion section.

## Discussion

The clinical impact of contrast-enhanced ultrasound imaging is growing rapidly, yet the application of qCEUS imaging in drug therapies remains limited. In this study, we combine qCEUS imaging with a technique known as “sonopermeation” (also called “sonoporation” or “sonopermeabilization”) to facilitate drug uptake in an experimental model of neuroblastoma and monitor tumor response to sonopermeation therapy *in vivo*. Sonopermeation using UCAs can effectively increase vascular permeability, thus eliminating dependence on the endogenous tumor vasculature. However, methods of monitoring and controlling vascular permeability *in vivo* are lacking. The overall goal of this study was to demonstrate that sonopermeation is a rational approach to improving the efficacy of liposomal doxorubicin therapy in neuroblastoma and that contrast-enhanced imaging can be used to monitor the effects of sonopermeation and accurately assess liposomal drug uptake *in vivo*. Over the course of this study, we developed a unique methodology with clinical potential for simultaneously monitoring and treating neuroblastoma (and other solid tumors) using sonopermeation therapy, capitalizing on 2D and 3D qCEUS imaging.

### Monitoring Sonopermeation and Evaluating Drug Uptake in Matrigel Plug Models

Our initial experiments were performed in Matrigel plug models in order to evaluate small molecule drug uptake (2 kDa) and liposomal doxorubicin uptake using relatively low-ultrasound intensities (<0.6 MPa). Our results demonstrated that sonopermeation did in fact promote small molecule delivery using 2 kDa FITC-Dextrans without noticeable changes in total perfusion (measured by 3D qCEUS imaging). Interestingly, we observed that reperfusion rates tend to increase following sonopermeation (94±57% with 10^8^ MBs; 47±9% with 10^9^ MBs; Figure 5B) and correlate with increasing drug uptake. The exact mechanism for improved perfusion rates (without changes in overall perfusion) in the Matrigel plugs following sonopermeation is unknown but may be attributed to pruning of the microvasculature and redirecting of blood to larger vessels more visible by ultrasound.

One of the key findings from our Matrigel study was that low-intensity ultrasound was able to facilitate small molecule drug delivery to Matrigel plugs *in vivo*, but not liposomal drug carriers (Figure 4C-D). We used high dosage chemotherapy (25 mg/kg doxorubicin) to facilitate visualization and detection of accumulated doxorubicin in these studies, however no increase in doxorubicin uptake could be observed with increasing ultrasound intensity. We rationalized that the encapsulation of doxorubicin into liposomes, which increases the overall drug size to ~80nm, likely requires higher levels of permeabilization to effectively extravasate into tissue, although other factors such as doxorubicin low quantum efficiency may also have resulted in poor detection in Matrigel plugs. In order to improve delivery, we sought to increase the ultrasound intensity applied during sonopermeation. The maximum output of the therapeutic transducer was ~0.6 MPa. To achieve higher ultrasound intensities, we developed an acrylic focusing lens that attached to the soundhead of the therapeutic ultrasound system (Supplementary Figure 1A). Using our focusing lens, we could apply higher amplitudes of pressure (up to 2 MPa) at the focus to the Matrigel plugs while imaging concurrently with a clinical scanner. Utilizing ultrasound as a guide to position the focus, we were able to demonstrate significantly enhanced levels of liposome uptake in the Matrigel plugs, using fluorescence microscopy of liposomes in sections of Matrigel plugs (Figure 5C). At our maximum power setting and high dosage of bubbles (1x10^9^ bubbles in 500 µL), the highest level of liposome delivery was observed without significant change in total perfusion following sonopermeation therapy (Figure 5A). This enhancement in liposomal uptake with increasing microbubble concentration is believed to be a consequence of higher degrees of vascular permeabilization. In Matrigel plugs, injections of both 10^9^ and 10^8^ MBs show increases in reperfusion rates (RR) after sonopermeation, however no significant difference *between* these two concentrations was observed. We attribute this to overall poor perfusion of Matrigel tumors which likely decreases sensitivity and increases variability in qCEUS imaging at lower MB concentrations. Since the change in RR was statistically significant at both microbubble concentrations, while no significant change in RBV was detected, we concluded that RR is a more sensitive measure of sonopermeation effect. It may be possible to improve accuracy using the area under the curve (AUC), which accounts for both RR and RBV terms in a single metric. This strategy has been used by similar groups 52 and will be pursued in future studies involving NGP tumors.

We then demonstrated that efficient L-DOX delivery could be attained using our focused ultrasound system. Doxorubicin measurements were taken in excised Matrigel plugs, liver, contralateral kidney, and heart tissue. Our results disclosed nearly a 5-fold increase in doxorubicin uptake in Matrigel plugs using focused ultrasound mediated sonopermeation (Figure 6), with no change in uptake in the heart. Therefore, it stands to reason that lower dosages could be applied to reduce overall cardiotoxicity while simultaneously improving intratumoral drug uptake.

### Monitoring Sonopermeation and Evaluating Doxorubicin Uptake in Neuroblastoma Models

Recognizing the limitations of the Matrigel plug model, the next phase of our project aimed to evaluate whether qCEUS imaging could effectively monitor sonopermeation in a highly relevant and well-characterized tumor model of human neuroblastoma. Similar to our previous results, TIC analysis illustrated that sonopermeated sections of NB tumors did not experience an overall decrease in their level of perfusion as compared to non-sonopermeated sections of tissue lying outside the tumor boundary (Figure 8A). This finding is inconsistent with the work of Burke *et al* 52 who reported significantly reduced perfusion following 60 min of sonopermeation therapy using an order of 10^6^ MBs at four different low duty cycle US pulsing schemes. Rather than relying on a tethered or co-injected chemotherapeutic agent to bring about anti-tumor effects, this group activated the tumor apoptotic pathway by provoking microvascular damage using microbubble cavitation. They reasoned that mechanical ablation due to ultrasonic microbubble destruction leads to reduced blood flow, which in turn diminishes tissue oxygenation and nutrient delivery, ultimately inhibiting tumor growth and lowering the number of viable tumor cells.

Identifying changes in tumor vasculature during sonopermeation is imperative, as blood vessels are the route of systemic chemotherapeutic delivery. Our results are particularly important as we can demonstrate that permeabilization of the vasculature is occurring without significant disruption of blood flow, which is crucial for increasing intratumoral drug penetration. We expect that increased levels of ultrasound exposure would eventually result in microvessel destruction or thermal ablation of tissue (which could also be measured by qCEUS imaging), but did not observe localized heating or tissue damage under the conditions we tested (Supplementary Figure 2).

Similar to earlier findings in Matrigel plug models, reperfusion kinetics of ultrasound contrast agents following flash destruction appeared to be a far more sensitive method of monitoring the effects of sonopermeation. However, contrary to our earlier findings, we observed a significant decrease in the rate of microbubble reperfusion (instead of increase) in sonopermeated tissue (Figure 8B, p<0.01) compared with unsonopermeated control tissue. This result, taken together with the evidence showing no change in relative blood volume of sonopermeated tumors (Figure 8A), indicates that vascular permeabilization was achieved without inducing blood vessel damage or destruction. We conjecture that the decrease in the rate of reperfusion is a direct result of increased hematocrit in the wholly intact blood vessels - that is, microbubble destruction effectively permeabilizes the vascular endothelium, causing plasma to leak out, thus raising viscosity within the lumen of the vessels. This in turn produces higher resistance to microbubble flow which is reflected in the qCEUS-derived time-intensity curve (TIC). To explore this phenomenon, we performed photoacoustic imaging to measure hemoglobin content within the tumor, before and after sonopermeation (Supplementary Figure 4). As hemoglobin is readily detectable and functions as an effective endogenous scatterer 53, hemoglobin was treated as a surrogate for hematocrit. Our preliminary results show an increase in hemoglobin content within the tumor immediately following sonopermeation, supporting the hypothesis that increases in hematocrit correlate with the observed increase in vascular permeability and improved drug uptake. Therefore, the biophysical effect that sonopermeation bears on microbubble perfusion kinetics - it appears to increase the fraction of red blood cells within vessels contained in the treated area - renders it an effective predictor of intratumoral drug uptake. No difference in oxygenated versus deoxygenated hemoglobin was observed. While this was only a single experiment, a recent report by Schultz *et al* 54 similarly demonstrated an increase in hemoglobin content in sonopermeated tumors using an analogous protocol, supporting our preliminary neuroblastoma data.

To establish a link between drug uptake and sonopermeation kinetics *in vivo*, L-DOX accumulation, changes in tissue morphology, and apoptotic biomarkers were evaluated. As depicted in Figure 9B, sonopermeation resulted in a 2.3-fold increase in L-DOX uptake in NB xenografts as compared to mice that were infused with microbubbles and L-DOX without focused ultrasound, and a 4.8-fold increase in uptake compared with untreated controls (Figure 9B). A decrease in the density of blood vessels was not observed in the sonopermeation group, ascertained by lectin staining (Figure 9A, middle row). This constitutes a critical finding as sonopermeation is intended to drive increased concentrations of drug into the tumor without triggering vascular collapse. Since therapeutic agents utilize the vasculature as a physical route to reach their target site, inflicting irreversible damage on the vasculature (endothelium and/or pericytes) would be detrimental to the goal of depositing large drug payloads into the tumor space, especially if repeated deliveries are to be attempted as required by most chemotherapy regimens. With this objective in mind, longitudinal studies investigating whether vascular changes associated with sonopermeation result in long-term remodeling (beyond 24 h) are still needed. In this particular body of work, we elected to only perform qCEUS during or immediately after sonopermeation and to evaluate changes in vasculature at 30 min and 24 h using *ex vivo* analysis; however, qCEUS imaging at extended time points in subsequent studies would more fully expound the tumor response to the increased doxorubicin payload.

We also sought to determine whether ultrasound-triggered microbubble destruction induces apoptosis by itself, that is, in the absence of L-DOX. H&E stained sections of sonopermeated tumors exhibited no changes in architecture or apoptosis (Figure 8F) immediately following sonopermeation, but immunostaining of the pericyte marker αSMA did reveal discontinuous coverage of pericytes encircling the vascular endothelium (Figure 8E). These changes in pericyte morphology suggest a mechanism for increased vascular permeability: loosening of interendothelial junctions, allowing greater extravasation. Future studies will address the longevity of these effects and explicate whether or not tight junctions are restored within 24 h.

### Broader Applications in a Clinical Setting

While the focus of this study was on neuroblastoma, we believe that the techniques outlined can have broader applications in a clinical setting for perfusion-guided drug therapies in a wide range of cancers. Here, we present a single case study from an ongoing clinical trial where sonopermeation was performed using simultaneous imaging and therapy with a protocol resembling that described in this paper. The procedure for sonopermeation can be found in the methods section and is summarized in Figure 10A, along with quantitative flash-destruction imaging (Figure 10B) and results (Figure 10C). It is important to note some of the key differences, which include microbubble concentration, microbubble type, and ultrasound application, due to stringent FDA regulations. Despite barriers in comparing directly between animal and human testing, this data serves as proof-of-principle that the strategy used in this paper can have direct clinical impact.

There are several challenges that still need to be overcome for human testing, including implementation of 3D imaging with motion correction to reduce variability in qCEUS imaging, and analysis of linearized data instead of logarithmically compressed video images. Importantly, real-time feedback to quantify reperfusion rates on-the-fly would be highly beneficial for evaluating sonopermeation efficiency during treatment. Additionally, more work needs to be done to correlate the qCEUS imaging parameters with long-term tumor response to therapy and overall survival.

## Conclusion

Quantitative contrast-enhanced imaging is attracting increasing interest, as UCAs are being employed for both diagnostic imaging as well as therapy. The applications of qCEUS imaging have the unique and inherent capacity to offer real-time assessment of therapeutic effectiveness. Monitoring tissue permeability of a drug, as well as controlling drug uptake with qCEUS, may dramatically improve the clinical outcomes of therapeutic interventions. As utilization of qCEUS imaging continues to advance in clinical settings, the techniques proposed here could become increasingly valuable, leveraging the growing variety of nanoparticle drug therapies aimed at varying tumor types in combination with emerging ultrasound imaging technologies.

## Supplementary Material

Supplementary methods and figures.

## Abbreviations

αSMA: alpha smooth muscle actin
BBB: blood brain barrier
CEUS: contrast enhanced ultrasound
CPS: contrast pulse sequencing
DiD: 1,1′-dioctadecyl-3,3,3′,3′-tetramethylindodicarbocyanine dye
EPR: enhanced permeability and retention
FD: flash-destruction
FITC: fluorescein isothiocyanate
FUS: focused ultrasound
Hb: hemoglobin
IGDD: image-guided drug delivery
IHC: immunohistochemistry
L-DOX: liposomal doxorubicin
MB: microbubble
NB: neuroblastoma
PA: photoacoustic
PFB: perfluorobutane
PnP: peak negative pressure
qCEUS: quantitative contrast enhanced ultrasound
RBC: red blood cell
RBV: relative blood volume
ROI: region of interest
RR: reperfusion rate
Sono: sonopermeation
TUNEL: terminal deoxynucleotidyl transferase dUTP nick end labeling
UCA: ultrasound contrast agent
US: ultrasound
UTMD: ultrasound-triggered microbubble destruction
