# Supplementary material for: Perfusion-guided sonopermeation of neuroblastoma: a novel strategy for monitoring and predicting liposomal doxorubicin uptake in vivo
Source: Theranostics. 2020 Jul 9;10(18):8143–61. doi: 10.7150/thno.45903 (PMC7381728; doi:10.7150/thno.45903)
Supplement: Supplementary file 1 — Supplementary methods and figures. [file thnov10p8143s1.pdf]

Supplementary Material

Focused Ultrasound Application

In order to generate focused ultrasound in this study, a custom focusing lens-and-cone was developed to fit on the soundhead of the 1 cm transducer attached to the SoundCare Plus therapy system (Figure S1A). The Acrylic stock (McMaster-Carr, Douglasville, GA, Product #8528K2) was cut with a laser cutter and milled with a high speed ½" steel ball end mill (Niagara Cutter P/N N24488) and polished with fine sand paper. The speed of sound was measured through the acrylic material at 2771 m/s. A custom coupling cone with inlets and outlets for water was 3D printed using an Objet350 Connex3 (Stratasys, Eden Prairie, MN printer with Vera Clear resin). The focal distance of the ultrasound beam was 3 cm. The pressure at the focus was measured using an Onda needle hydrophone (Sunnyvale, CA, HNC-0200) with attached pre-amplifier (AH-2010-DCBNS). The soundfield was mapped in 2D at the focal length using a custom MATLAB 3D acquisition system (Figure S1B) at a 0.1 W/cm^2 output, the lowest intensity of the SoundCare Plus therapy system. The peak-negative pressures were recorded from 0.1-3 W/cm^2 at a 3 cm distance and are reported in Figure S1C. The maximum output pressure without the lens was 0.88 MPa peak-negative-pressure (PnP), while the maximum output pressure with the lens was 2.35 MPa (PnP). It should be noted though that when the sonoporator was used without the attached lens-and-cone system, the emitting ultrasound disc transducer was placed directly on the animal where the near-field pressures were observed in some areas to reach upwards of ~1.26 MPa (data not shown). It is therefore likely that some microbubbles experienced higher pressures than reported in S1C without the lens during sonopermeation.

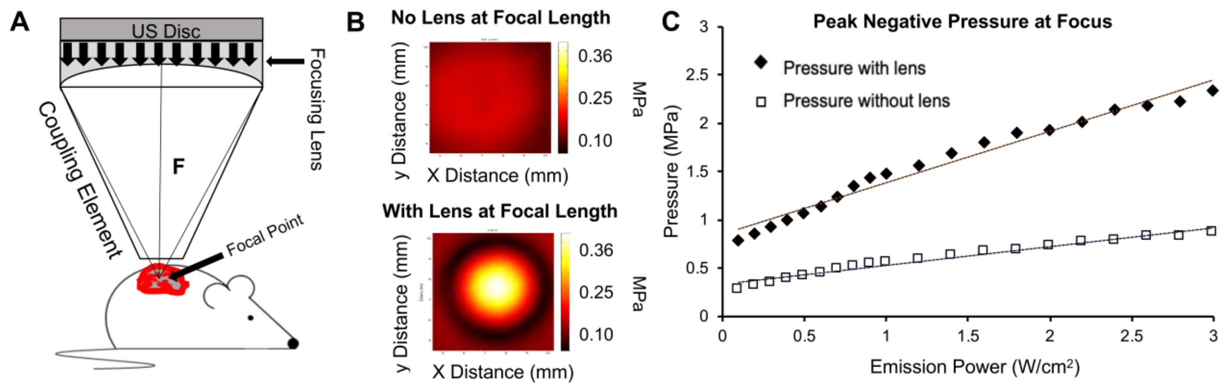

Supplementary Figure 1. (A) A custom focusing lens was developed for the sonoporator to increase the ultrasound intensity in the tumor. (B) A 2D map of the focal zone was performed using an Onda Hydrophone at the lowest output from the sonoporator (0.1 W/cm^2) to confirm effective focusing of the ultrasound. The focusing lens allows better spatial control of contrast agent destruction *in vivo* within the tumor. (C) The pressure output from the sonoporator shows significantly higher ultrasound intensity at the focus with increasing sonoporator output. We would like to acknowledge Dr. Paul Mountford for design and assembly of the lens-and-cone system. We would like to acknowledge Dr. Jake Dove and Dr. Todd Murray at the University of Colorado Boulder for providing the 2D mapping of the soundfield.

In Vitro Temperature Monitoring

To quantify heat generated by the application of focused ultrasound, the handheld therapeutic transducer with attached lens-and-cone was focused onto an acoustically transparent pipette bulb filled with 4 mL of deionized water (Figure S2A). The transducer was powered at different intensities and duty cycles for a period of 3 min and recorded signals (sampled every 100 ms) were inputted to a commercial thermocouple-to-analog converter (AD Instruments). The amplified output was then passed through a DAQ integrated with the custom LabVIEW software program. Noise reduction was accomplished by taking a rolling average of the prior 30 samples in MATLAB. As reflected in Figure S2B, a temperature increase of less than 1 °C was noted with 3 W/cm^2 at 10% duty cycle, signifying that the vascular changes observed at these *in vivo* operating parameters are not due to thermal effects but rather to bubble cavitation effects. At the same power, 30% duty cycle caused the temperature to climb 2.5 °C, 50% triggered a rise of 5 °C, and 70% elicited a temperature spike of 7 °C.

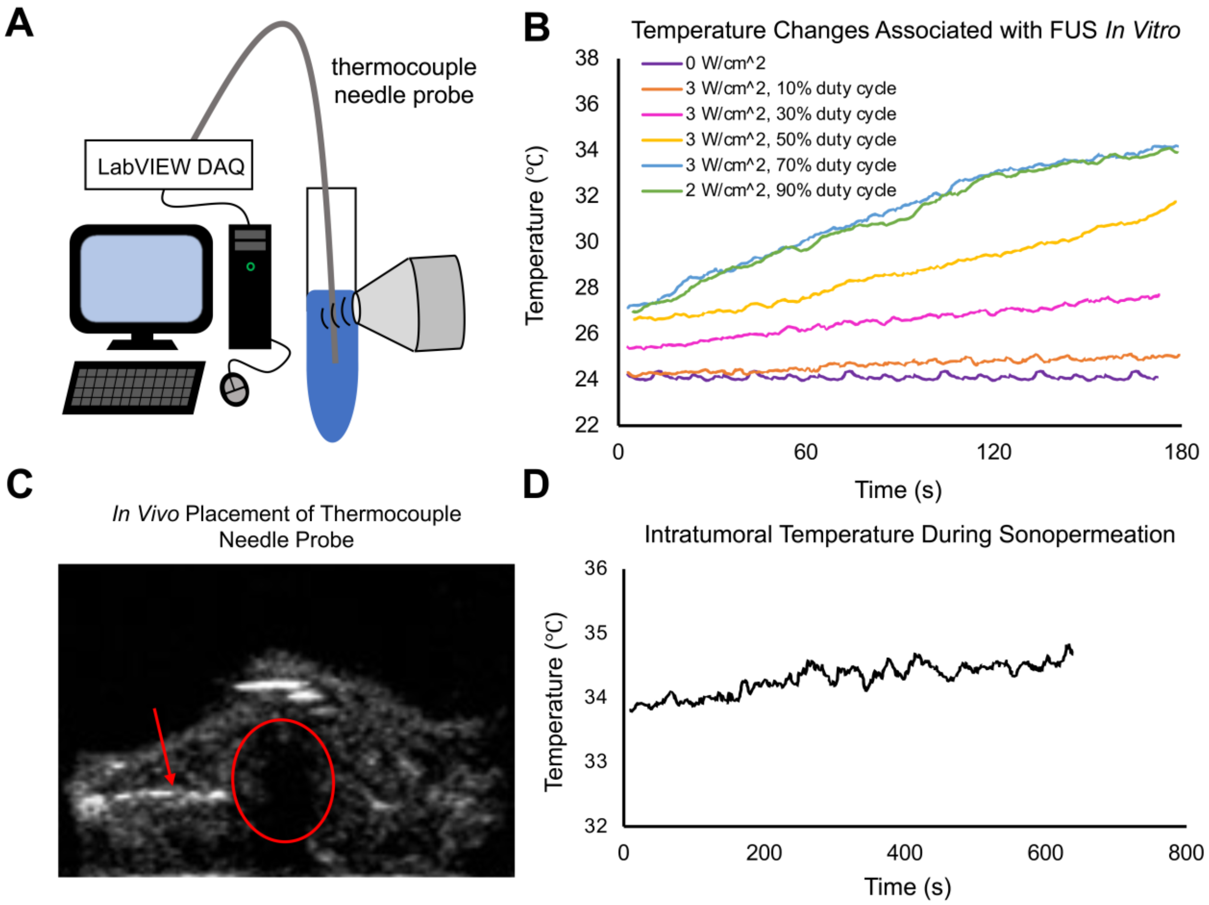

Supplementary Figure 2. (A) Cartoon graphic of experimental setup for *in vitro* temperature monitoring study. (B) Temperature elevations plotted for different sonoporator output parameters. (C) Screen capture from clinical scanner showing the insertion of a microneedle thermocouple probe into the sonopermeation focal zone of an NGP xenograft. Intratumoral temperature during sonopermeation increases by less than 1 °C (D) but sonopermeation dramatically increases vascular permeability.

In Vivo Temperature Monitoring

An NGP-bearing mouse (n =1) was sonopermeated as described in the methods section. Whole body temperature was maintained at 37 °C using a closed loop temperature control system comprising a heat lamp and a rectal probe (Physitemp Instruments, Clifton, NJ). A 0.64 mm flexible type T thermocouple microprobe (AD Instruments) was inserted into the tumor via an 18-gauge guide needle and positioned such that the probe tip extended into the focal zone, as marked in Figure S2C. Intratumoral temperature measurements were collected for the duration of the 10 min sonopermeation experiment and for an additional minute following the final flash-destruction

pulse. High frequency noise was smoothed by computing a rolling average of the preceding 100 samples using the same MATLAB script employed in the *in vitro* temperature experiment. The resultant temperature curve plotted in Figure S2D reveals that temperature elevation was limited to 1 °C, suggesting that the increased blood flow and vascular permeability observed during sonopermeation are a consequence of microbubble-mediated mechanical effects rather than the induction of local hyperthermia.

RBC Extravasation in Matrigel Plugs With and Without Focused Ultrasound

Red blood cell extravasation was evaluated in histology sections with both unfocused and focused ultrasound application at 2W/cm^2. Implantation of Matrigel plugs was performed as described in the methods of the main manuscript. Sonopermeation and excision of tumors was performed using similar techniques as described in the IHC analysis of tumors, although Matrigel plugs were not perfused in these experiments. Excised plugs were fixed in PFA following removal and sectioned for H&E staining. Example H&E stains are shown in Figure S3.

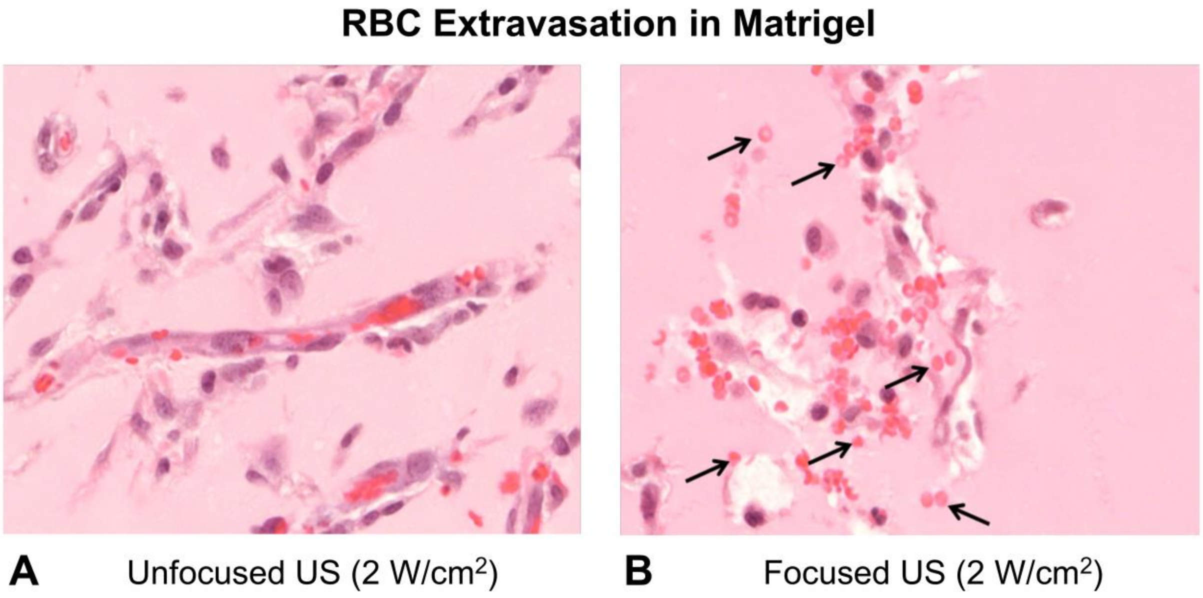

Supplementary Figure 3. Example H&E sections of Matrigel Plugs (A) with unfocused ultrasound and (B) focused ultrasound application. Following unfocused application at 2W/cm^2 (corresponding to ~0.7 MPa PnP), RBCs appear to be confined to the blood vessels, whereas significant extravasation was observed (indicated by arrows) with focused ultrasound at 2W/cm^2 (corresponding to ~2 MPa PnP in the -6dB focal zone).

Similar observations of RBC extravasation are observed for NGP tumors that have been perfused (Figure 8D) following sonopermeation although no increases in overall perfusion were observed during qCEUS imaging.

Photoacoustic Imaging

2D photoacoustic imaging was performed pre- and 30 min post-sonopermeation (n =1) using a Vevo 3100 LAZR scanner equipped with a LZ250 PA probe (Fujifilm Visualsonics, Toronto, Canada). After orienting the transducer longitudinally above the largest cross-section of the tumor, the laser was fired at 100% power at wavelengths of 750 and 850 nm with a PA signal gain of 40 dB. During analysis, tumor boundaries were outlined manually in the VevoLAB user interface for quantification of the PA signal over the delineated area (Figure S4). The software's built-in measurement package was used to determine total hemoglobin signal (HbT); this metric is linearly proportional to the sum of the oxygenated and deoxygenated hemoglobin concentration. HbT values were averaged over 30 frames and a comparison of the pre- and post-sonopermeation HbT Avr values showed a ~9% increase in hemoglobin content following sonopermeation (Figure S4). Although only a single tumor was evaluated in this study, Schultz *et al* [54] found that both hemoglobin and oxygenation levels increased detectably in 10 mice bearing pancreatic ductal adenocarcinoma (PDAC) xenografts following sonopermeation.

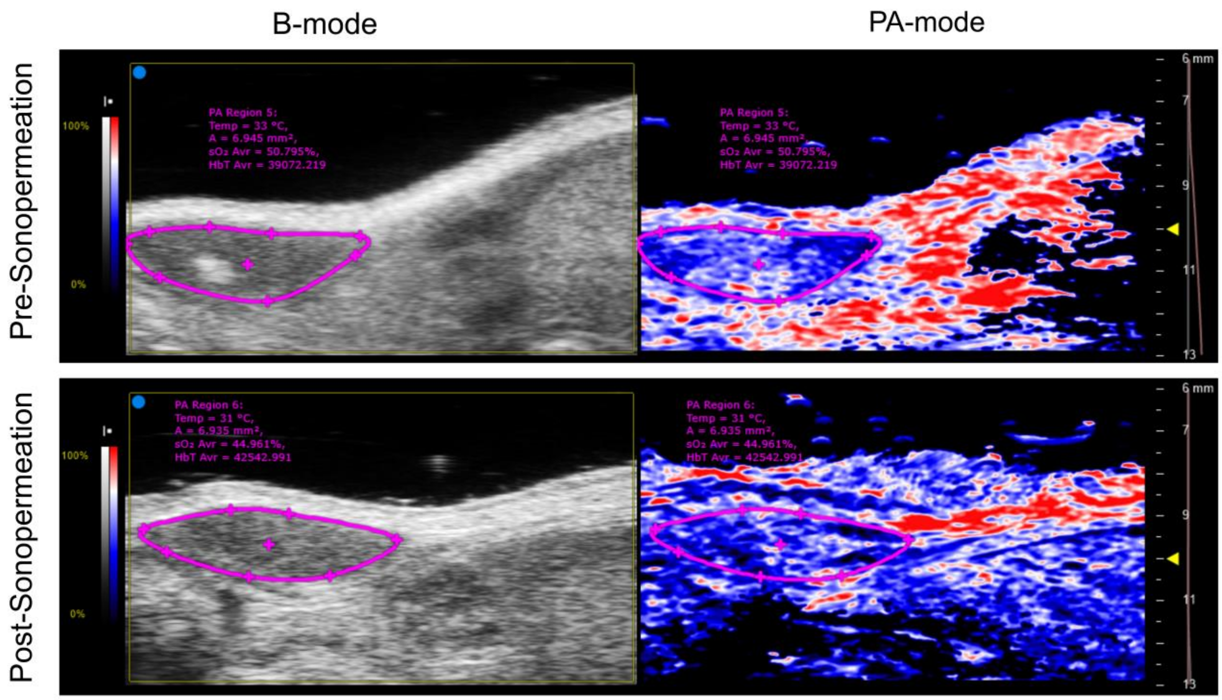

Supplementary Figure 4. Comparison of tumor hemoglobin levels at baseline prior to sonopermeation and 30 min after completion of sonopermeation treatment. The image depicts an overall increase in total hemoglobin from 39702 pre- sonopermeation to 42543 post- sonopermeation in the chosen imaging plane, which co-registers with the largest cross-sectional diameter of the tumor.

Effect of Sonopermeation on Blood Vessel Lumen Diameter

Vascular caliber (blood vessel diameter) was performed on chromogenic Endomucin stained whole tissue scans measured on Pannoramic Viewer (3DHISTECH). Untreated tissue was compared to treated NGP tissue at 30 min and 24 h post-sonopermeation. We defined the vascular caliber as the longest axis of the inside of Endomucin staining, using the "Create Distance Measurement Annotation" on Pannoramic Viewer. One hundred measurements were created from each tumor section, three tumors were analyzed per group, and statistical analysis performed in Prism (GraphPad) using a two-way ANOVA, two-tailed exact test. Lectin- and αSMA-positive cells were quantified from fluorescent images taken at 10x magnification on an Axioscop2 (Zeiss), and using the threshold tool in ImageJ. Statistical analysis was performed using Excel (Microsoft) with Wilcoxon Signed Rank Test. Four pictures per tissue were taken from fresh frozen samples, excluding kidney areas and non-viable tumor areas, and three tumors per group were analyzed.

Overall, significant increase in blood vessel lumen diameters were observed at both 30 min and 24 h time points post-sonopermeation. Vessel caliber quantification at 30 minutes was performed only using two tumors.

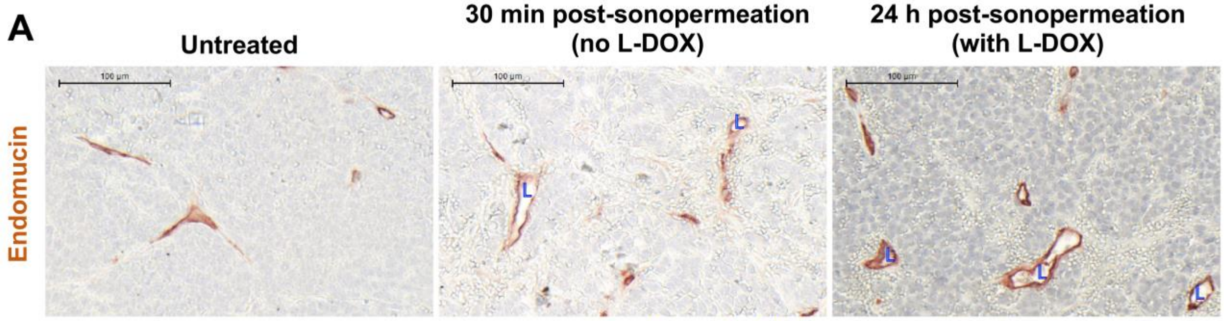

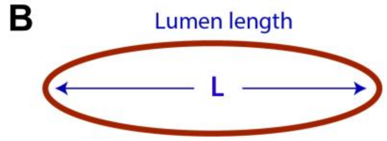

C

	Lumen length Median	[IQR]	Rank Sum	n	p-value
Untreated	15.89	[10.99, 27.26]	653	3 (102)	
30 minutes	21.54	[14.96, 31.51]	3003	2 (102)	< 0.0001
24 hours	29.79	[16.84, 44.68]	4072	3 (102)	< 0.0001

Supplementary Figure 5. Sonopermeation enlarges blood vessel lumens in neuroblastoma xenografts. (A) Representative images of Endomucin immunostain (brown color) in untreated tumors (left panel), 30 min after sonopermeation without L-DOX (middle panel), and 24 h after sonopermeation with L-DOX (right panel). Lumens are marked with a letter L (blue). (B) Diagram illustrating lumen length measurements: blood vessel lumens were demarcated by endothelial marker Endomucin (brown) and measured along their longest axis (blue arrows). (C) Table summarizing median lumen length, [interquartile range (IQR)], sum of signed ranks, number of animals (number of pictures per tissue), and two-tailed p-values after Wilcoxon Signed Rank Test of the three tested groups.
